# CBX3:IL1RN Reflects Distinct Cellular States That Defines the Clinical Outcome of Oral Squamous Cell Carcinoma

**DOI:** 10.1002/cam4.71705

**Published:** 2026-03-09

**Authors:** Xutengyue Tian, Jixiong Mao, Dongguo Li, Zhengxue Han, Qiaoshi Xu

**Affiliations:** ^1^ Department of Oral and Maxillofacial & Head and Neck Oncology, Beijing Stomatological Hospital Capital Medical University Beijing China; ^2^ School of Biomedical Engineering Capital Medical University Beijing China

**Keywords:** CBX3:IL1RN, CMS subtype, epithelial‐mesenchymal transition, gene pair, oral squamous cell carcinoma

## Abstract

**Background:**

Oral squamous cell carcinoma (OSCC) exhibits heterogeneous therapeutic outcomes owing to various tumor microenvironments (TMEs). Previous studies have classified OSCC according to its molecular characteristics; however, clinical practice is limited by technical complexity.

**Methods:**

A total of 33 patients from five single‐cell datasets of primary OSCC were integrated for CMS classification. The correlation between CMS classification and prognosis was analyzed with integrated bulk transcriptomics. Top scoring pairs (TSPs) algorithm was used to identify the gene pair that effectively captures the information of CMS classification. The selected gene pair was ultimately validated in independent single‐cell data, spatial transcriptomics and immunofluorescence assays.

**Results:**

OSCC patients were stratified into two distinct molecular subtypes (CMS1 and CMS2) harboring diverse prognostic levels, therapeutic vulnerabilities, microenvironmental characteristics, and malignant behaviors. Furthermore, CBX3 and IL1RN were identified as signature genes whose expression ratio (CBX3:IL1RN) effectively captures the critical features of CMS classification. Patients whose CBX3:IL1RN > 1 exhibited characteristics of CMS1 subtype with worse outcomes and more active malignant behaviors, vice versa. Further analyses implicated epithelial‐mesenchymal transition (EMT) as a potential mechanism through which this ratio may modulate tumor progression. Finally, our finding was validated with our clinical samples and confirmed the ratio of CBX3:IL1RN was related to T and N stages.

**Conclusion:**

This study presents a simplified and clinically applicable OSCC classification system based on CBX3:IL1RN. And provide a practical tool for OSCC subtyping with implications for prognosis prediction and personalized treatment strategies.

## Graphical abstract

Previous classification is limited in clinical practice for complex gene signatures, technical procedure and expenses. We proposed CBX3:IL1RN as a simple gene pair marker to stratify patients into CMS1 and CMS2 subtypes. This classification can not only reflect the prognosis but also assist therapeutic plan.

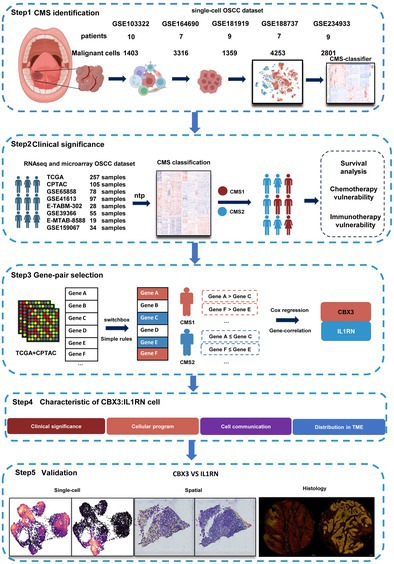

**Abbreviations**
CMS: consensus molecular subtype
CNV: copy number variation
DEG: differentially expressed gene
EMT: epithelial‐mesenchymal transition
GRN: Gene Regulatory Network
HVG: highly variable gene
NMF: non‐negative matrix factorization
OS: overall survival
OSCC: oral squamous cell carcinoma
PD‐1: programmed cell death protein‐1
PD‐L1: programmed cell death ligand‐1
TME: tumor microenvironment
TPM: transcript per million
TSP: top scoring pair
UMI: Unique Molecular Identifier

## 1 Introduction

Oral squamous cell carcinoma (OSCC) is a common neoplasm of the head and neck. With fine surgical treatment and neoadjuvant therapy, the survival rate can only reach about 50% [1], let alone the trauma OSCC brings to patients physically and mentally. In clinical practice, patients with the same clinical stage show varied responses to identical therapeutic approaches [2]. It illuminates hope for precision oncology, which urges us to identify more accurate stratification of patients with OSCC. To date, the varied vulnerabilities and prognosis of OSCC are believed to be associated with the complexity and heterogeneity of the TME [3].

Several studies have attempted to elucidate the TME based on its molecular characteristics. Seminal studies using RNA‐seq data and single‐cell data have classified cancer into diverse subtypes [4, 5, 6, 7]. These molecular classifications demonstrated good performance in predicting prognosis. However, most gene signatures proposed in the prediction models can hardly be implemented into clinical practice, due to the following reasons. Firstly, the evident cost and requirement for quality control impede transcription utilization in clinical use. Secondly, the complex signatures proposed by varied studies are often inconsistent, owing to diverse platforms and processing methods. Finally, few signatures can assist clinical actions in formulating therapeutic plans [8, 9].

To identify reliable and practical markers for OSCC classification and prognosis prediction, a robust and simple classifier is required. The gene pair classifier is advantageous in this regard. This approach leverages the relative expression of gene pairs rather than an extensive gene panel. Classification is performed simply “within” the expression profile of a single patient unrelated to the whole cohorts [10, 11, 12]. With this top scoring pair (TSP) algorithm, chances are provided to identify simple, interpretable, and clinically significant biomarkers.

Bulk transcriptomics was once prominent for molecular subtyping, but with low resolution [13, 14]. Single‐cell transcriptomics enable us to uncover distinct roles of different cell populations. Nevertheless, it regards cells of patients rather than patients themselves as units, hindering formal testing to inter‐tumor variation between patients. In this study, we utilize pseudo‐bulk profiles to focus on the inter‐tumor variation and discovered CMS1/CMS2 in OSCC, harboring varied biological behaviors, microenvironments, vulnerabilities, and clinical outcomes. More importantly, to enhance the clinical relevance of our classification, we identify a simple gene pair (CBX3 and IL1RN) that reflects the clinical and biological traits of CMS1 and CMS2. Moreover, we validate these results in histopathological analysis. The ratio of CBX3 to IL1RN has demonstrated significant clinical value that links to neoadjuvant sensitivity and early neck dissection necessity.

## 2 Materials and Methods

### 2.1 Data Collection

Primary OSCC single‐cell RNA sequencing (scRNA‐seq) data were retrieved from five Gene Expression Omnibus (GEO) datasets: GSE103322, GSE164690, GSE181919, GSE188737, and GSE234933 for downstream classification analysis [5, 15, 16, 17, 18]. Bulk RNA‐seq data with primary OSCC samples were retrieved from The Cancer Genome Atlas (TCGA) and the Clinical Proteomic Tumor Analysis Consortium (CPTAC). Additional microarray profiles, including GSE65858, GSE41613, GSE39366, GSE159067, E‐TABM‐302, and E‐MTAB‐8588 [19, 20, 21, 22, 23], along with associated clinical information, were retrieved from GEO and EMBL's European Bioinformatics Institute(EMBL‐EBI) for validation. An independent OSCC single‐cell dataset (GSE215403) was retrieved from GEO for external validation [24]. GSE251902 was retrieved to investigate the interaction between fibroblasts and malignant cells [25]. The spatial transcriptome data of GSE208253 were retrieved to support our finding [26]. The detailed information about online available data is provided in Table S1.

### 2.2 Data Pre‐Processing and Filtration

Data preprocessing followed established platform‐specific protocols. All this scRNA‐seq data were then processed using the Seurat R package for downstream analysis and visualization [27]. For bulk RNA‐seq data (TCGA‐HNSC, CPTAC‐HNSC, and GSE159067), raw count data and transcript per million (TPM) data were acquired for further analysis. Raw microarray data were pre‐processed according to each platform. Affymetrix data (GSE41613 and E‐TABM‐302) were pre‐processed with affy package [28]. Illumina data (GSE65858 and E‐MTAB‐8588) were pre‐processed via lumi package [29]. Agilent data (GSE39366) was pre‐processed using limma package [30].

### 2.3 Identification of Malignant Cells

To distinguish malignant epithelial cells from their normal counterparts, copy number variations (CNV) were inferred from scRNA‐seq data using the InferCNV package [31]. Scaled Unique Molecular Identifier (UMI) count data from different cohorts were extracted, pooled, and processed using inferCNV. CNV values were then clustered using Seurat. Patient‐specific CNV values were calculated as the median of the CNV values of malignant cells. Using patient‐specific CNV values, we were able to categorize patients through hierarchical clustering.

### 2.4 Regulons Analysis

Gene Regulatory network (GRN) inference was performed using the PySCENIC package [32]. scRNA‐seq data from five OSCC malignant epithelial cell cohorts were used as the inputs. GRNboost2 was used to construct the GRN. Regulon activity was calculated using AUCell. The AUC values were then pooled and scaled for clustering and differential analyses using Seurat.

### 2.5 Subclustering of Malignant Cells

To define malignant epithelial subclusters, informative gene sets were identified using the DUBStepR algorithm [33]. Highly variable genes (HVGs) from GSE234933 were selected for subclustering to better capture biological differences [34]. Malignant cell clustering utilized the top feature genes with num.pcs = 20 and min.cells set to 5% of all cells, while other parameters remained default. Differential expression analysis was conducted using the FindMarker function in Seurat to identify cluster‐specific differentially expressed genes (DEGs), defining the top 30 upregulated and downregulated DEGs as pairwise markers. Malignant cells were re‐clustered with Seurat at a resolution of 0.5. Genes with absolute log2 fold‐change > log2(1.5) and adjusted *p*‐value < 0.05 (Bonferroni) were classified as CMS, yielding 971 genes.

We then averaged the transcriptomes of malignant cells from each cohort within each patient to create pseudo‐bulk data. Each data was visualized by performing PCA based on 971 genes as features. Silhouette scores, gap statistics, elbow method, consensus clustering and hierarchical clustering were used to determine the possible number of subtypes. In order to minimize the batch effect, we zero‐centered and scaled each gene to unit variance before integration. The integrated data was processed as data from individual cohorts. And similar process was done to determine possible subtype number. Eventually, we clustered integrated pseudo‐bulk data using hierarchical clustering.

### 2.6 Pseudotime Trajectory Analysis

We used the Monocle 2 R package to infer the potential developmental lineages of CMS malignant cells. The CMS classifier was used to order cells along a pseudotime axis, with all other parameters set to default. The Cytotrace2 R package was then used to infer the developmental potential and ensure the trajectory root.

### 2.7 Final Marker Gene Selection for CMS Clustering

To identify the final set of subcluster‐specific marker genes, pseudo‐bulk expression matrix was calculated by summing the batch‐corrected counts (zero‐centered and scaled) from five single‐cell transcriptomics. We then used DESeq2 for batch correction and to identify the final markers. lfcShrink was performed Genes with FDR < 0.05, Log2FoldChange > 1or < −1were selected as the final CMS markers (139 genes).

### 2.8 CMS Classification of Bulk Transcriptome

Eight bulk tumor transcriptomes were compiled and individually analyzed to assign tumor samples to CMS subtypes. The top 50 markers of CMS1 and CMS2 were defined as the reference templates. The expression values of bulk transcriptomics were processed using the nearest template prediction (ntp) algorithm implemented in CMScaller [35, 36] to characterize the patients for CMS classification; FDR < 0.05 was set to restrict prediction error rate. The SubCamera algorithm was used to compare the pathway activities of CMS1 and CMS2.

### 2.9 Analysis of DNA Methylation and Mutation in OSCC

The SNP and DNA methylation data for OSCC samples were obtained from the TCGA‐HNSC cohort via the TCGAbiolinks. Somatic mutation profiles were analyzed and visualized using the maftools package with oncoprint plots. GISTIC2.0 was used on GenePattern to detect CNVs.

### 2.10 Deconvolution of TME

The Bayesprism R package was used to infer the composition of TME from bulk transcriptomics [37]. We used annotated single‐cell datasets from GSE103322 for testing and GSE234933 for validation to define cell types. TCGA count data were used as the bulk data. Default settings were applied for the analysis.

### 2.11 Cell–Cell Communication Analysis

The R package CellChat (v1.5.0) was used to analyze the cell–cell communication between malignant and non‐malignant cells [38]. Downstream processing followed the default CellChat pipeline. The NicheNet (v2.2.0) R package was used to infer differential ligand–target interactions between CMS1 and CMS2 [39]. Malignant cells were defined as “receiver cells, and all other cells were defined as ‘sender cells’”. For DE analysis, clustered cells with gene expression over 5% were considered.

### 2.12 Top Scoring Pair Analysis

The SwitchBox R package, a rank‐based top‐scoring pair classifier [12], was used to select gene‐pair markers. The TCGA and CPTAC count data were integrated to create the training matrix. The previous CMS classifications of TCGA and CPTAC were used as training groups. Forty gene pairs were calculated as top scoring pairs (TSPs) for CMS classification.

### 2.13 Survival Analysis

Survival analysis was conducted on bulk transcriptomic datasets with available follow‐up data using the survival and survminer R packages. Kaplan–Meier analysis was performed to evaluate the association between CMS classification and patient prognosis.

### 2.14 Patients and Specimens

A total of 72 patients diagnosed with OSCC who were treated in the Department of Oral and Maxillofacial Head and Neck Oncology, Beijing Stomatological Hospital, Capital Medical University, were enrolled in this study. All enrolled patients were diagnosed with OSCC by biopsy at the outpatient clinic and were not subjected to any treatment prior to resection of the primary tumor. Tissue samples were obtained immediately after complete tumor removal. The medical records of the enrolled patients were collected after discharge for further analysis. HNSCC cases were staged according to the 8th edition of the Union for International Cancer Control/American Joint Committee on Cancer (UICC/AJCC) classification system. The baseline demographics of the enrolled patients are summarized in Table S2.

### 2.15 Multiple Immunofluorescences Staining and Analysis

To avoid non‐specific staining of antibodies from the same species, multiple immunofluorescence staining was performed to those 72 samples (61 patients with sufficient clinical information) using the TSA Plus kit based on tyramine signal amplification techniques according to the manufacturer's instructions. Primary antibody CBX3 (abcam, ab213167, dilute 1:100), and IL1RN (proteintech, 10844‐1‐AP, 1:100) were incubated overnight at 4°C, HRP oat anti‐Rabbit IgG (Servicebio, GB23303, 1:500), HRP Goat anti‐Mouse IgG (Servicebio, GB23301, 1:500) incubated for 60 min at 37°C. Spontaneous fluorescence was removed from paraffin sections using a tissue spontaneous fluorescence quencher immediately after antigen retrieval. All tissue slices were scanned with a Pannoramic Scanner using Pannoramic DESK, P‐MIDI, and P250 (3D HISTECH, Hungary).

### 2.16 Colocalization Analysis and Scoring

The colocalization analysis was conducted using the package Colocalization Finder, and the settings were left in default. The staining intensity was scored as follows: 0, negative absence of stained cells; 1, weak; 2, moderate; and 3, strong. The relative expression score was calculated by multiplying the area (percentage) of positive cells by staining intensity. Scoring was conducted by researchers who were blinded to the group information of tumor samples or the clinical data of the patients.

### 2.17 Statistical Analysis

All statistical analyses were performed in R (v 4.4.0), with the exception of the colocalization analysis, which was performed using ImageJ. Wilcoxon rank‐sum tests were used for noncategorical values. Student's *t*‐tests were adopted to compare the infiltration of different cell populations and predicted drug sensitivity boxplot. The chi‐square test was used to compare the associations between CMS classification and drug sensitivity. Spearman's correlation was used to exhibited the similarity among subtypes and the expression relationship among gene pairs. Log‐rank test was used for survival analysis. Wald tests were used in cox regression. For multiple testing corrections, we applied the Bonferroni method specifically during CMS classifier feature selection to reduce false positive outcomes. In all subsequent analyses, the false discovery rate (FDR) method was implemented. Two‐tailed *p* values of < 0.05 was considered statistically significant.

## 3 Results

### 3.1 CMS1 and CMS2 Are Two Distinct Molecular Subtypes of OSCC

**FIGURE 1:**
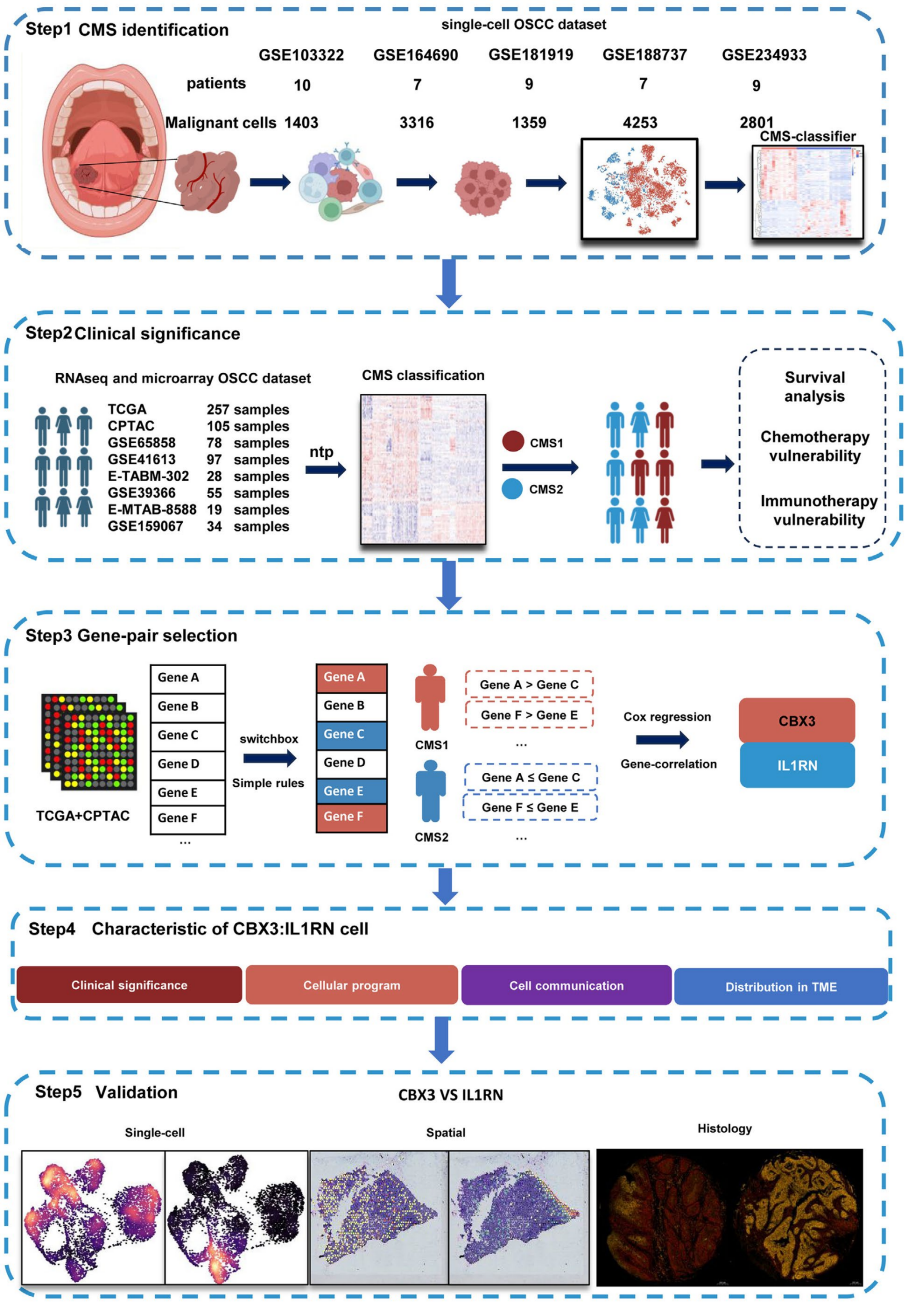
Study flowchart and analytic pipeline. Five OSCC single‐cell transcriptomes and eight bulk RNA transcriptomes were included in this study. The number of patients and malignant epithelial cells profiled in each dataset is indicated. The major bioinformatics analysis and experiments were conducted following this flowchart.

The main workflow of our study is illustrated in Figure 1. We used single‐cell datasets to define the CMS classifier. Bulk RNA datasets were then integrated to check the clinical significance of the CMS classification. To simplify the classification, a TSP algorithm was utilized to select the critical gene pair that could properly represent the clinical trait and cellular status of CMS. CBX3:IL1RN was selected and validated via independent single‐cell, spatial, and histological analysis.

**FIGURE 2:**
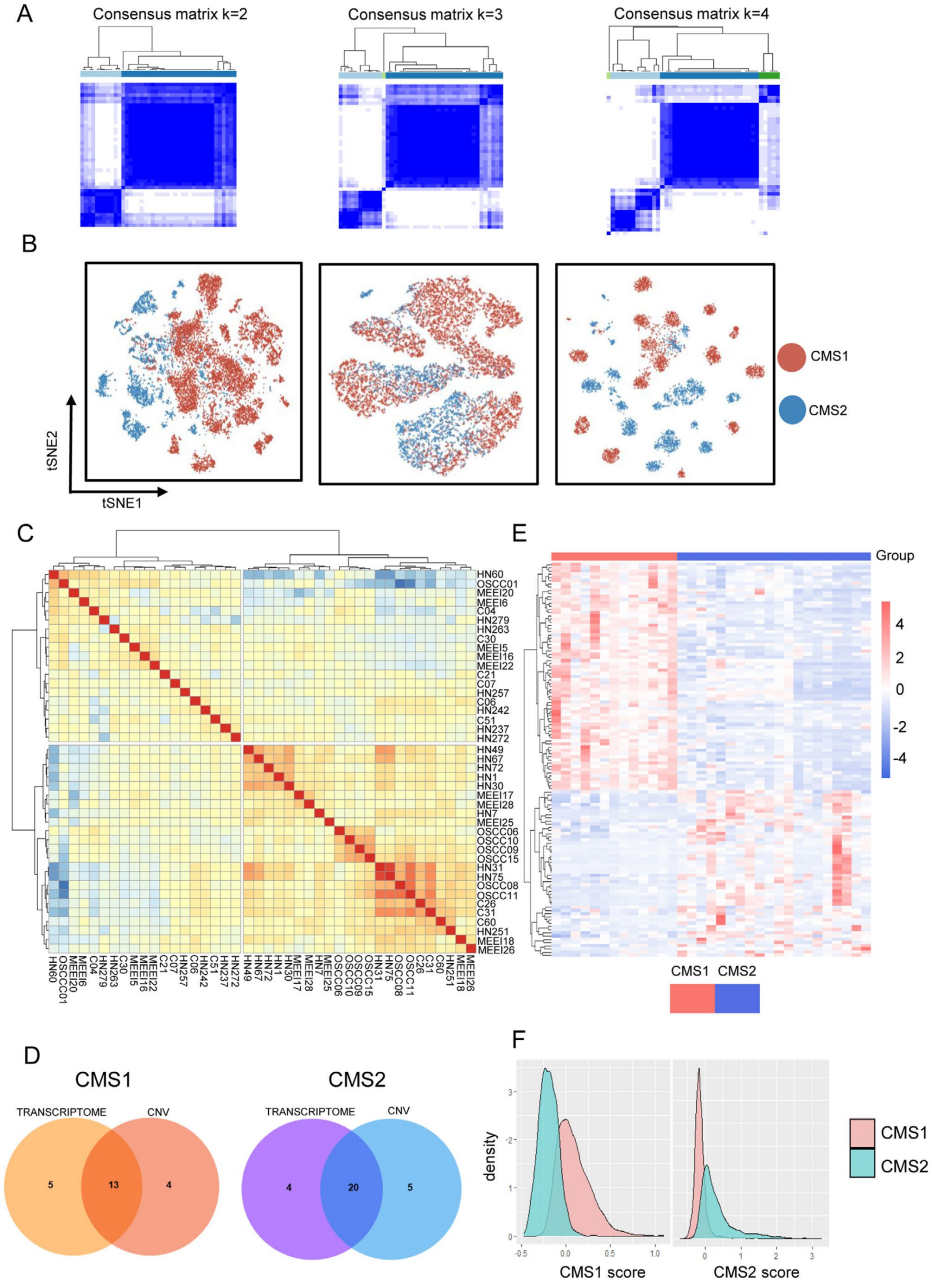
CMS1 and CMS2 are two distinct subtypes of OSCC. (A) Consensus clustering of an integrated single‐cell RNA dataset that reveals 2 as the appropriate number for classification. (B) TSNE plots display the clustering of single‐cell data from OSCC malignant cells based on three different classifiers. Clustering based on transcriptome data (left); clustering based on the AUC activity (middle); clustering based on CNV values (right). (C) Correlation (Spearman's correlation) heatmap indicates the high similarity of patients in the same CMS subtype from the pooled cohorts. (D) Venn diagram illustrates the patients who are consistently classified across both transcriptome and CNV profiles. (E) Heatmap visualizes pseudo‐bulk data of OSCC malignant cells, highlighting the final set of CMS markers as its features. (F) Density plot displays the module scores for the final CMS1 and CMS2 markers.

To explore the diversity of tumors among patients Given that malignant cells are the primary drivers of tumor progression and with obvious heterogeneity, we focused our analysis on this population. After the initial quality control, cell annotation, and infiltration. Five single‐cell datasets were included. Those five datasets are comprised of sufficient untreated primary OSCC samples with high data quality (Figure S1A). Among them, the primary OSCC samples of GSE234933 were retained for preliminary classification (Figure S2B). The 2801 malignant cells were extracted, followed by the DUBStepR algorithm and DEG analysis to identify 971 genes (CMS classifier). Malignant cells were clustered again based on the CMS classifier (Figure S2B). Since scRNA‐seq data treat individual cells, rather than patients, as units of analysis, pseudo‐bulk aggregation was used to capture inter‐patient differences for classification. Unsupervised consensus clustering and hierarchical clustering confirmed “two” as the number of subtypes with better performance based on the CMS classifier. Malignant cells were successfully separated into two fractions by PCA (Figures S2C,D and S3E). Then we integrated all the pseudo‐bulk data from five single‐cell OSCC datasets (42 patients with 13,132 cells) and clustered them using the CMS classifier. Similarly, “two” was most supported across datasets (Figure 2A,C, Figure S2A–E,H). In single‐cell resolution, to reduce the batch effect single‐cell data was zero‐centered and scaled before integration. The kBET test results indicated a closer alignment between the expected and observed rejection rates following our “scale” integration (Figure S2F) [40]. The score calculated by Seurat‐Integrate package indicated the better performance of our “scale” integration compared to Combat and Harmony (Figure S2G) [41]. The integrated data was visualized in the t‐SNE map based on 971 features, where CMS1 and CMS2 showed proper separation (Figure 2B left).

To identify the patient‐to‐patient genomic diversity of malignancies, we used inferCNV to infer the copy number variants (CNVs) and adopted the pseudo‐bulk CNV profiles for hierarchical clustering (Figure S2H,J). Here, patients with consistent category to CMS classification were retained for further analysis. Thirty‐three patients were retained (13 patients with CMS1 and 20 patients with CMS2) (Figure 2D, Figure S2F,H). In CNV profiles, CMS2 showed frequent deletions in chr3, chr7, and chr8 and frequent gains in chr11 and chr16. CMS1 showed gains on chr3, chr7, and chr8 (Figure S2I). We investigated transcriptional regulation differences using pySCENIC to infer regulon activity scores for clustering malignant cells from 33 patients. This analysis revealed distinct separation of regulon activity and CNV of malignant cells at single‐cell resolution based on CMS grouping (Figure 2B middle and right).

To acquire a robust classification, we obtained 139 marker genes by pooling all pseudo‐bulk profiles of 33 patients and then performing DEG analysis with the Deseq2 R package (Figure 2E, Table S3). The distribution of CMS metagene scores revealed that CMS classification may represent two distinct core components of OSCC rather than a continuous process (Figure 2F, Figure S3A–E).

### 3.2 Characteristics of CMS in Single‐Cell OSCC Samples

**FIGURE 3:**
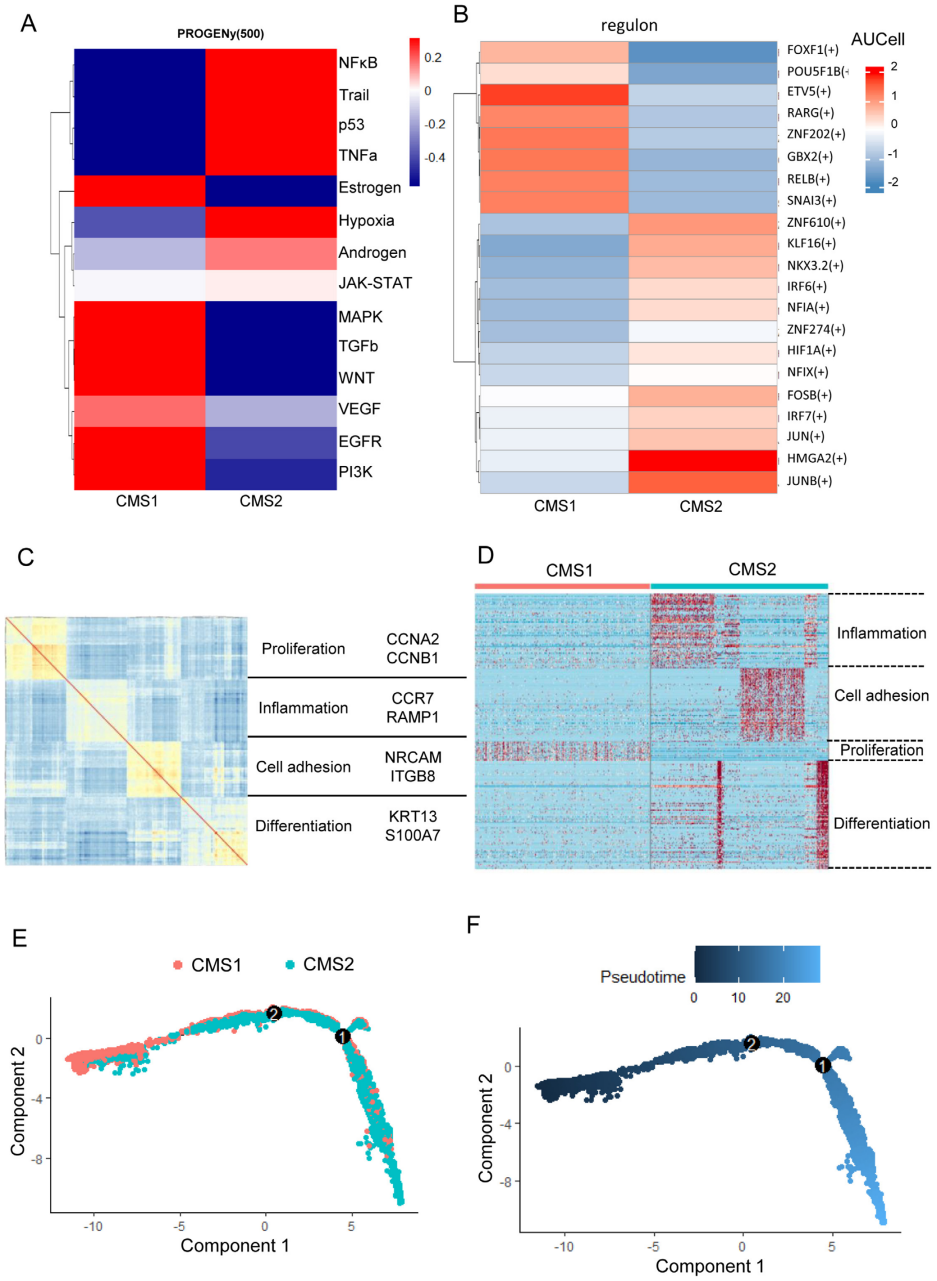
Cellular states of CMS1 and CMS2 in malignant cells. (A) Heatmap displays different pathway activities (using top 1000 expressed genes from single‐cell transcriptomes) among CMS inferred by PROGENy algorithm. (B) Heatmap displays significant CMS‐specific regulons, whose activity is calculated through AUCell algorithm. (C) Heatmap illustrates the correlation of genes identified through cNMF (axes indicate genes); the values reveal correlation coefficient. (D) Heatmap reveals distinct enrichments: CMS1 is characterized by proliferation‐associated genes, whereas CMS2 is enriched with genes related to inflammation, differentiation, and cell adhesion (each row represents z‐scored gene expression). (E, F) Pseudo‐time of malignant cells, and each point reflects a single cell. Colored by CMS classification and pseudo‐time.

We next aimed to investigate the functional and developmental diversity of the CMS1 and CMS2 subtypes. PROGENy inferred that CMS1 displayed elevated EGFR, TGF‐β, and estrogen pathways, while NFκB, TNFα, and androgen were enriched in CMS2 (Figure 3A). Transcription factor analysis revealed upregulation of ETV5 and SNAI3 in CMS1 malignant cells, suggesting activation of the EMT program in CMS1 (Figure 3B). We performed non‐negative matrix factorization algorithm (NMF) on malignant cells to explore the diversity in biological functions, by which identified four modules representing proliferation, inflammation, cell adhesion, and differentiation [42]. CMS1 carcinoma cells were only featured with proliferative function (Figure 3C,D), while CMS2 carcinoma cells exhibited features of inflammation, cell adhesion and differentiation. To assess the developmental potential and stemness of malignant cells, we performed pseudotime trajectory analysis using Monocle and Cytotrace2 algorithms. The results indicated that CMS1 cells were closer to the starting point, suggesting their progenitor‐like state, while CMS2 cells were more differentiated (Figure 3E,F, Figure S3F–J). These findings indicated that the CMS comprised two distinct cellular states involved in diverse signaling pathways and functions.

### 3.3 Clinical and Genomic Characterization of CMS OSCC Bulk Samples

**FIGURE 4:**
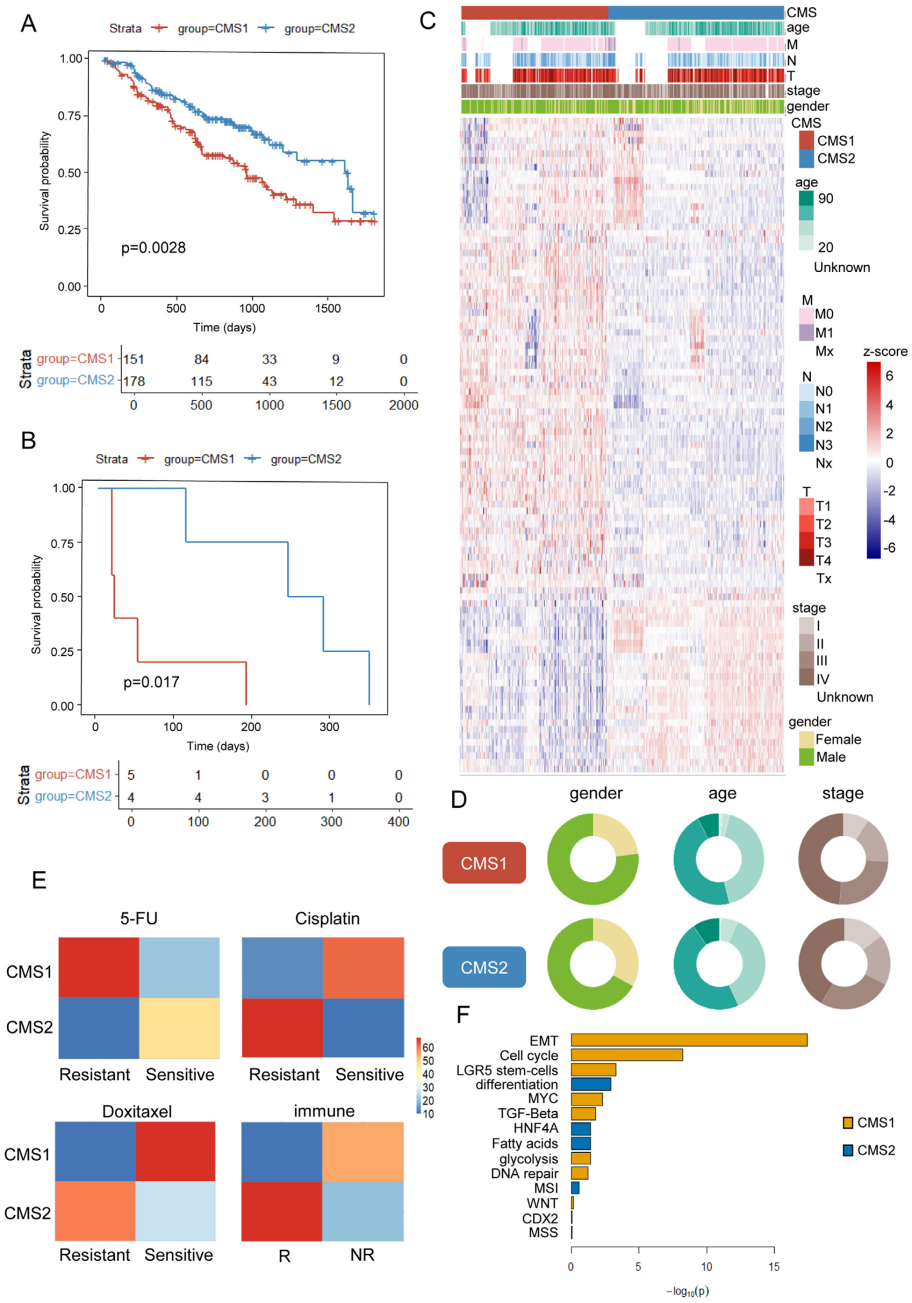
Clinical characteristics of CMS1 and CMS2 mapped to bulk datasets. (A) Kaplan–Meier survival curves are presented for integrated bulk transcriptomes from various datasets, distinguishing between CMS1 and CMS2 subtypes. (B) Kaplan–Meier survival curve is depicted for the GSE159067 cohort, which includes patients undergoing PD‐1/PD‐L1 targeted therapy. The patients have been classified into CMS1 and CMS2 subgroups. (C) Heatmap of scaled integrated bulk transcriptomes including GSE159067 and GSE39366. Clinical characteristics (gender, age, TNM stage and pathological stage) are also indicated. (D) Pie graphs show the differences in age, gender and stage between CMS1 and CMS2. (E) Heatmaps illustrate the sensitivities of CMS1 and CMS2 to various drugs and immunotherapies, with colors representing the number of patients. (F) Bar plot shows the differentially activated hallmarks of the CMS1 and CMS2 bulk transcriptomes.

Next, we tried to determine if there was clinical significance in the CMS classification. We pooled the bulk transcriptomics of five datasets (TCGA, CPTAC, GSE65858, GSE41613, E‐TABM‐302) and robustly classified them using ntp with 139 marker genes mentioned above (Figure S4A–G). Samples were stratified into CMS1 and CMS2. Patients who were classified as CMS1 displayed poorer 5‐year overall survival (OS) than those classified as CMS2 (Figure 4A). In addition, CMS2 patients receiving immunotherapy displayed better prognosis in 1‐year OS than CMS1 patients, demonstrating better response to PD‐1/PD‐L1 inhibitors (Figure 4B). The link between the CMS1 subtype and both male sex and advanced stage—factors associated with poor prognosis—aligned with our clinical observations. However, age seemed to be irrelevant to CMS classification (Figure 4C,D).

We next investigated treatment vulnerabilities of CMS subtypes using the OncoPredict package. The publicly available cell line dataset GDSC2 was used to predict the drug sensitivity of CMS patients. CMS1 cells showed significant resistance to 5‐FU but were sensitive to cisplatin and docetaxel (Figure 4E). TIDE (Tumor Immune Dysfunction and Exclusion) was used to predict the response to immunotherapy. We found that CMS1 patients were non‐responsive to immunotherapy, whereas CMS2 patients showed a positive response (Figure 4E). The SubCamera algorithm in the CMScaller package suggested that EMT might be a critical trigger for such differences in clinical characteristics (Figure 4F).

**FIGURE 5:**
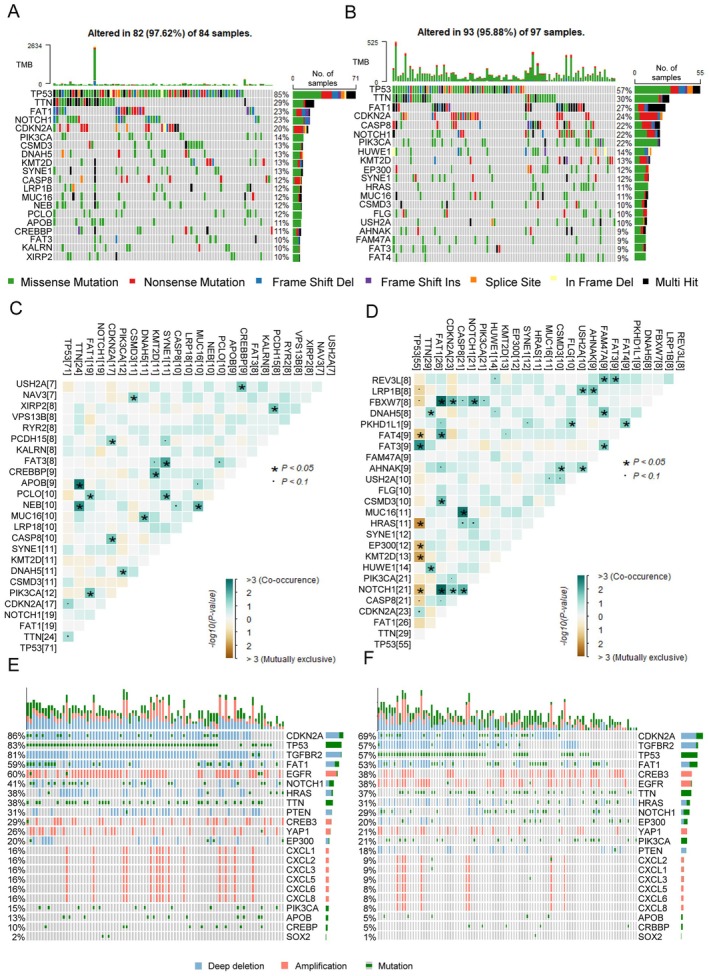
Gene alterations of CMS1 and CMS2 patients. (A and B) Oncoprints illustrate the mutational differences between CMS1 and CMS2 within TCGA SNP datasets. (C and D) Heatmaps depict the correlation patterns between mutated genes in CMS1 and CMS2. (E and F) Oncoprints of CNV profiles of CMS1 and CMS2 inferred through GISTIC.

We further assessed genomic differences between CMS1 and CMS2. CMS1 was found to be associated with increased TP53 alterations (Figure 5A,B). In contrast to patients assigned to CMS2, in whom TP53 mutation was negatively correlated with critical malignant progression factors (HRAS, EPHA3, and NOTCH1), no such correlations were observed in the CMS1 patients (Figure 5C,D). CNV analysis revealed obvious EGFR amplification and PTEN deletion in CMS1 (Figure 5E,F), which could partly explain the poor prognosis in CMS1 in the background of genetic mutation. Together, these findings demonstrated that CMS classification was an innate and intrinsic characteristic of OSCC.

### 3.4 TME Characterization Among CMS

**FIGURE 6:**
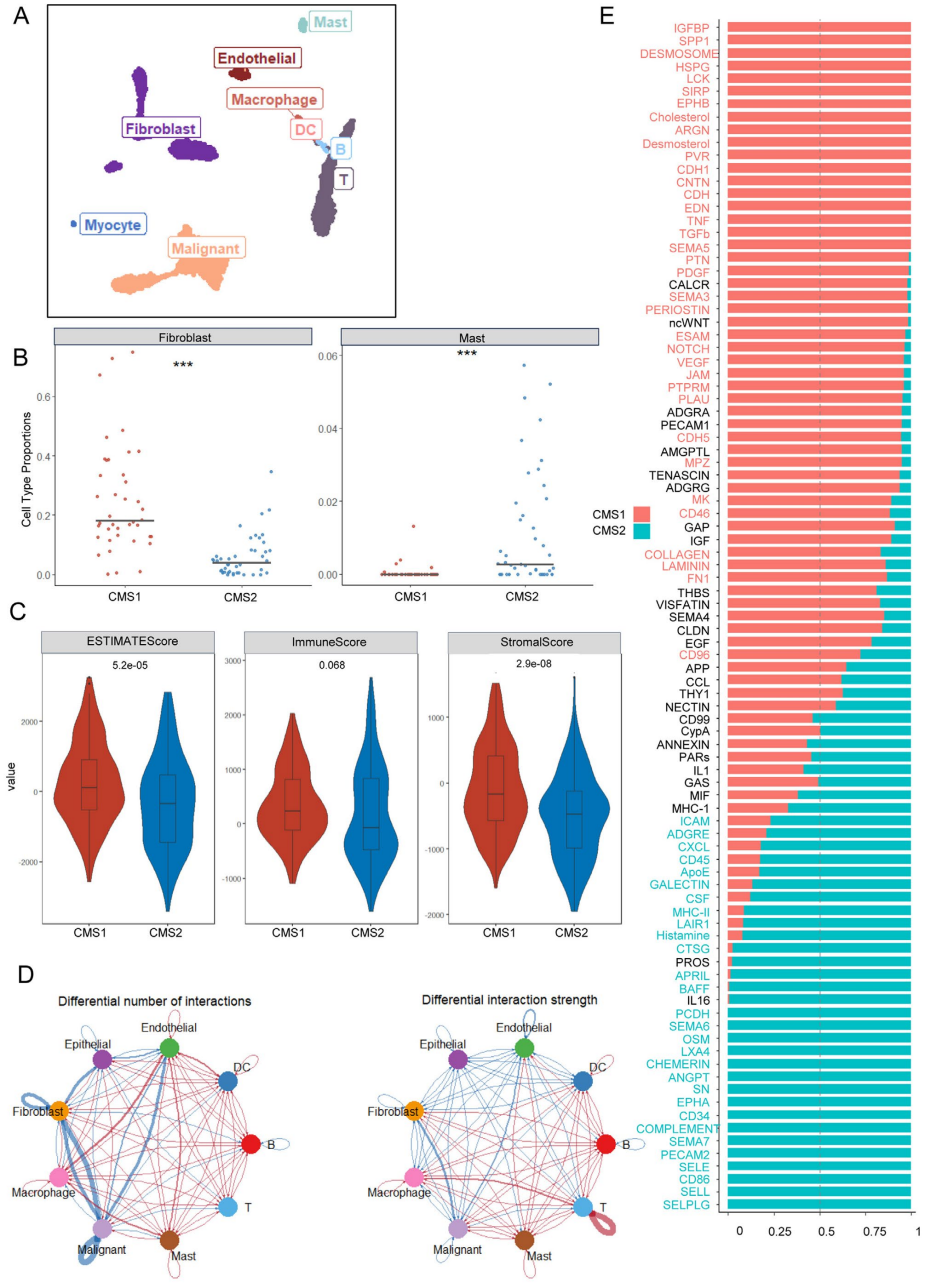
The construction and cell‐communication in TME of CMS1 and CMS2. (A) UMAP plot of CMS assigned OSCC patients in GSE103322. The major cell types are then annotated. (B) Plot visualizes the significant infiltration of fibroblasts in CMS1 and mast cells in CMS2 through the integrated analysis GSE103322 and TCGA using Bayesprism. (C) Violin plot that illustrate inferred ESTIMATE, immune, and stromal scores. (D) Chord graphs display the differential interactions between CMS2 and CMS1 based on CellChat analysis. The blue line highlights a decrease in interactions in CMS2, whereas the red line emphasizes an increase in interactions within CMS2, differential weights of these interactions are also indicated. (E) The receptor interaction rank based on NicheNet analysis through comparison between CMS1 and CMS2. Gene names highlighted in red indicate dominant interaction in the CMS1 associated tumor microenvironment. While gene names highlighted in blue indicate dominant interaction in CMS2.

Next, we attempted to determine how malignant cells in different CMS subtypes shaped the TME and reversely influenced themselves. Using GSE103322 as a reference, we deconvoluted bulk transcriptomic data to estimate the infiltration of various cells (Figure 6A). The proportion of fibroblasts and mast cells differed among CMS. CMS1 was endowed with more fibroblast infiltration, whereas CMS2 was endowed with mast cells (Figure 6B). In parallel, GSE234933 proved the robustness of the results (Figure S5A,C) and further discovered abundant neutrophil infiltration in CMS2 (Figure S5C). The ESTIMATE demonstrated stromal infiltration of CMS1, leading us to focus on the interactions between malignant cells and fibroblasts (Figure 6C). Of note, weaker interactions between malignant cells, endothelial cells, and fibroblasts were found in CMS2 comparing to CMS1 (Figure 6D, Figure S5B). In CMS1, growth factors (e.g., IGFBP1) along with SPP1, an established marker of poor prognosis, were significantly enriched. In CMS2, the inflammation‐related factors CXCL, MHC‐II, and SEMA7 were enriched (Figure 6E). NicheNet results revealed TGFB1 as the top‐ranked ligand activity (Figure S5D–G), suggesting CMS1 tumors fostering a fibroblast‐rich, growth factor‐enriched microenvironment conducive to epithelial‐mesenchymal transition (EMT) activation.

### 3.5 CBX3 and IL1RN Identify CMS Classification of OSCC

**FIGURE 7:**
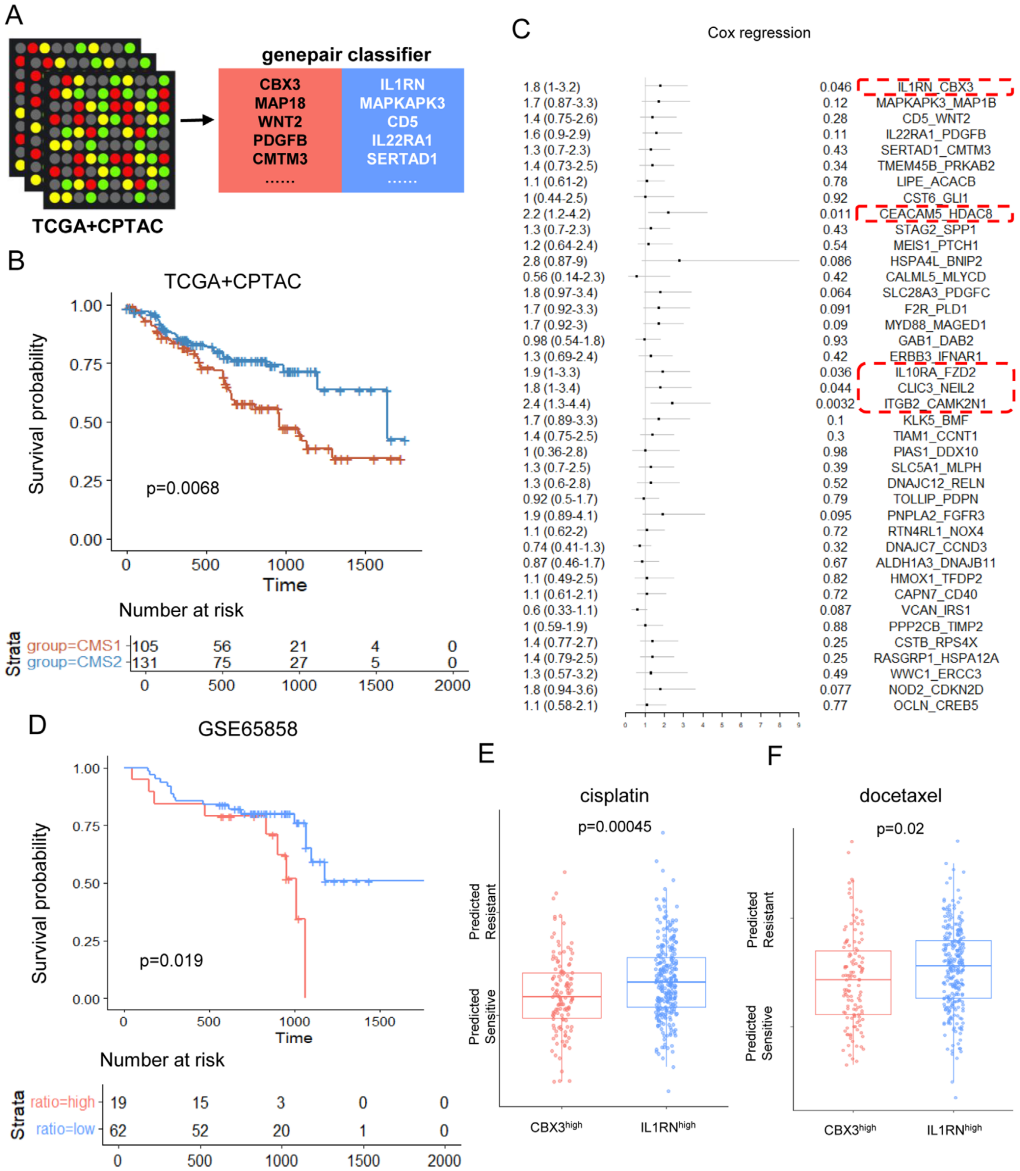
CBX3:IL1RN determines the CMS classification and clinical outcome of OSCC. (A) Graph displays gene pairs selected via Switchbox by utilizing the pooled TCGA + CPTAC data. (B) Kaplan–Meier curve illustrates the survival outcomes of CMS1 and CMS2 patients, identified through gene pair classification, utilizing data from TCGA and CPTAC. (C) Summary table of Univariate Cox Regression Analysis for Gene Pairs in TCGA + CPTAC Dataset. (D) Kaplan–Meier survival curves validate the survival rates of patients with high CBX3:IL1RN ratio and low CBX3:IL1RN ratio in the GSE65858. (E, F) Boxplots show the varied predicted chemosensitivity values to cisplatin and docetaxel among CBX3^high^ patients and IL1RN^high^ patients (cutoff = 1). The y‐axis indicates predicted IC50 by OncoPredict R package.

Here, we sought to acquire a simpler classification. We exploited the Switchbox package, a top‐scoring gene‐pair algorithm, to simplify the CMS classification. Twenty‐three gene pairs were recognized to simulate CMS classification (Figure 7A). ROC curves showed AUC scores of both internal validation and external validations were above 0.9 (Figures S6A and S7A). Survival analysis demonstrated the same result to CMS classification, among patients receiving standard therapy or immunotherapy (Figure 7B, Figure S6B). To identify the key gene pair. Cox regression was conducted. Gene pairs associated with prognosis (e.g., IL1RN and CBX3) were retained (Figure 7C). Since a limitation of the TSP is its ignorance to absolute gene expression, we additionally investigated the expression relationship of each gene pair. Ideally, the genes in a pair should be negatively correlated to better reflect the polarity of CMS1/CMS2. Only IL1RN and CBX3 met this criterion in both RNA‐seq data and microarray data (Figure S6C,D). CEACAM5:HDAC8, IL10RA:FZD2, CLIC3:NEIL2 and ITGB2:CAMK2N1 did not show a significant and consistent negative correlation across cohort (Figure S7A,B). Thus, IL1RN and CBX3 (ranked first in gene pairs) were selected for subsequent analyses. Of note, the methylation data demonstrated that the negative correlation between CBX3 and IL1RN might be the result of CBX3 methylation regulation, which was also the reason for EMT activation (Figure S8D,E).

To validate the rationality of classifying patients based on CBX3 and IL1RN expression, we included all the samples from the bulk datasets to find CBX3:IL1RN association with survival (Figure 7D, Figure S8B,C). In addition, the CBX3:IL1RN ratio could also predict the response to immunotherapy, cisplatin, fluorouracil, docetaxel, and cetuximab. We defined CBX3^high^ (CBX3:IL1RN > 1) as CMS1‐like, IL1RN^high^ (CBX3:IL1RN ≤ 1) as CMS2‐like. IL1RN^high^ patients were sensitive to immunotherapy (Figure S6E). CBX3^high^ patients were predicted to be sensitive to cisplatin and docetaxel (Figure 7E,F), while unsensitive to fluorouracil and cetuximab (Figure S6F). Thus, the CBX3:IL1RN ratio could serve as a robust, clinically translatable biomarker for CMS classification and prognosis prediction in OSCC.

### 3.6 CBX3 and IL1RN Represent Two Distinct Cellular State

**FIGURE 8:**
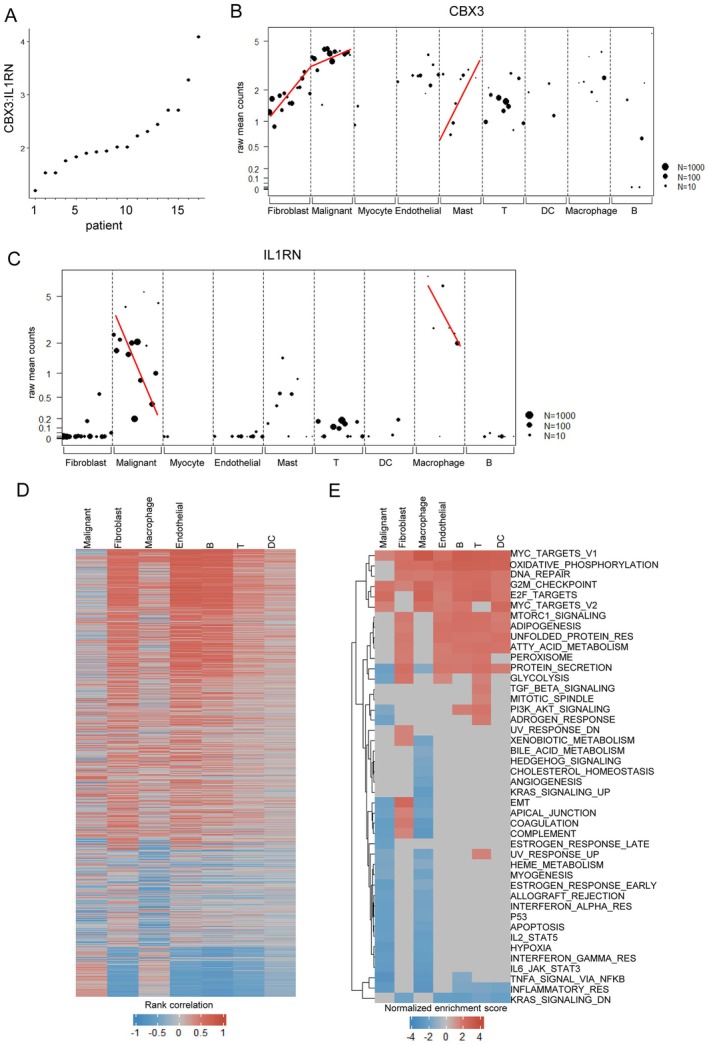
CBX3 and IL1RN determine varied TME and cellular pathways. (A) Scatter plot illustrates the ranking of patients from the GSE103322 dataset (17 tumors), based on their CBX3:IL1RN ratio. Each dot signifies an individual patient. (B, C) Scatter plot shows the expression levels of CBX3 and IL1RN in the major cell types. Patients are ranked from the lowest to the highest CBX3:IL1RN ratio. Spearman's rank correlation is used and indicated with a red line. (D) Heatmap displays the correlation of genes with the CBX3:IL1RN ratio across various cell types. Genes that are significantly correlated (*p* < 0.05, log2FoldChange > 0.5) with CBX3 are indicated in red, while those correlated with IL1RN are indicated in blue. (E) Heatmap of enriched hallmarks of these correlated genes in different cell types. Pathways that are significantly correlated with CBX3 and IL1RN are presented.

We next investigated the distribution of CBX3 and IL1RN among different cell populations. CBX3 was widely expressed by both malignant and non‐malignant cells. For IL1RN, only malignant cells, mast cells, T cells, and macrophages significantly expressed. We ranked patients based on CBX3:IL1RN (Figure 8A) and found that CBX3 from fibroblasts, malignant cells, and mast cells, and IL1RN from malignant cells and macrophages were co‐regulated with the ratio (Figure 8B,C). GSEA analysis revealed that CBX3 across all cell types was associated with the Myc pathway and oxidative phosphorylation, while IL1RN correlated with the KRAS pathway and inflammatory response. Notably, EMT was affected by IL1RN in malignant cells and macrophages and CBX3 in fibroblasts, encouraging the results that EMT was not regulated solely by either CBX3 or IL1RN (Figure 8D,E). Study on co‐cultured malignant cells and fibroblasts showed that CBX3 was linked to ligand‐receptor interactions, extracellular matrix degradation (e.g., MMP15), and chemokines (e.g., CXCL8), while IL1RN primarily correlated with inflammatory factors like IL1A and TNF (Figure S9A–D). Coordinately, CBX3:IL1RN conducted pro‐ or antitumor signals that regulated cellular programs and states of the TME, which may ultimately result in varied clinical outcomes.

**FIGURE 9:**
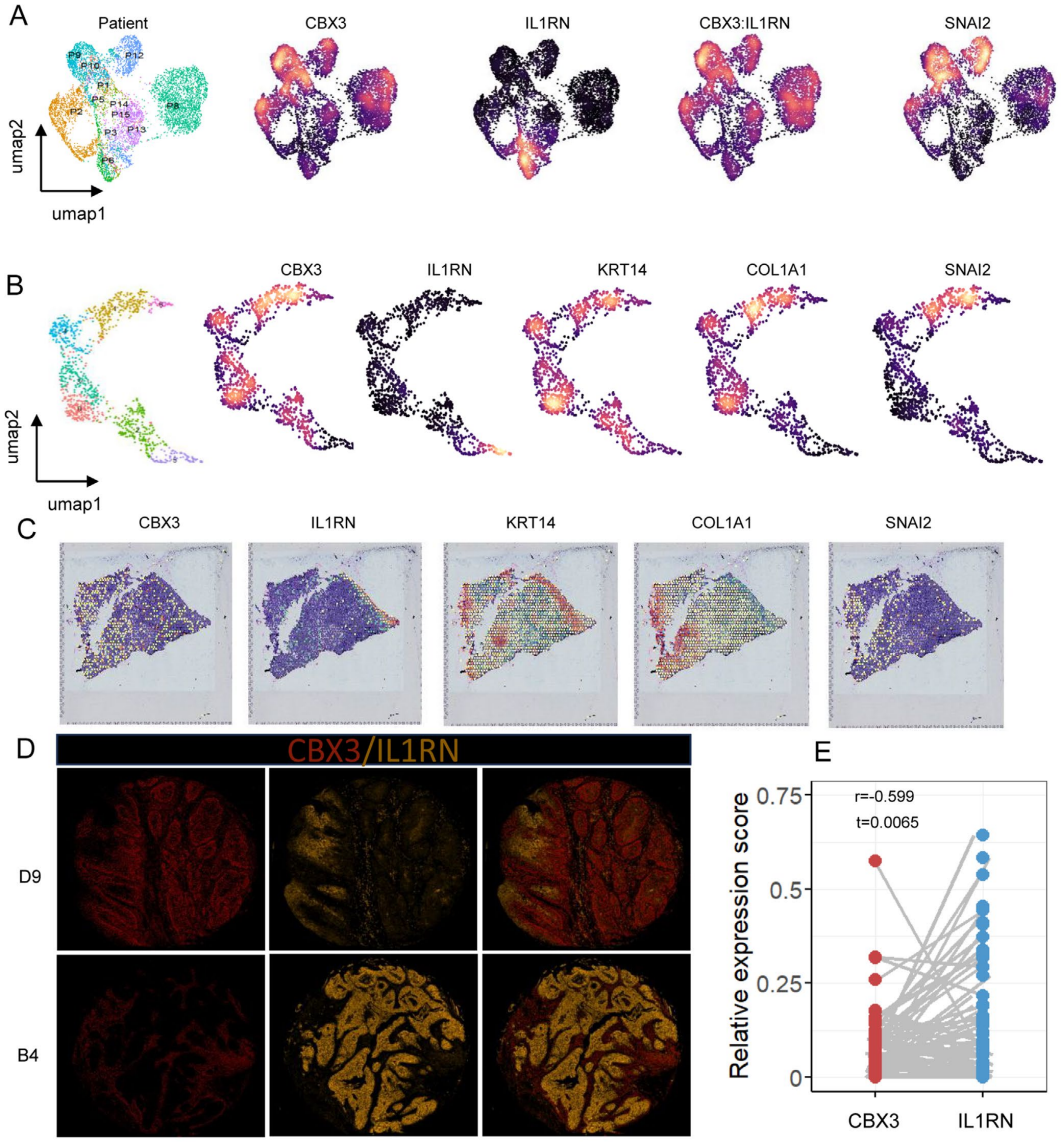
CBX3 and IL1RN are distributed in a patient‐specific and spatially distinct manner. (A) UMAP plots visualize OSCC malignant cells in GSE215403. Patients ID, CBX3, IL1RN, SNAI2, and CBX3:IL1RN ratio are denoted. (B, C) UMAP plots and spatial plots of spatial transcriptome GSE208253 show (sample 1) the expression of CBX3, IL1RN, KRT14, COL1A1 and SNAI2. (D) Multiple immunofluorescence images show the distribution of CBX3 (red) and IL1RN (yellow) in patients label with D9 and B4. D9 represents (CBX3 > IL1RN) and B4 represents (IL1RN < CBX3). (E) Plots exhibit the correlation of CBX3 and IL1RN in Multiple immunofluorescences. Each plot represents a tissue sample.

To validate our findings, we utilized independent single‐cell transcriptome and spatial transcriptome. CBX3 and IL1RN were observed to be opposingly expressed in individuals (Figure 9A). Even within the same tissue section, the spatial distributions of CBX3 and IL1RN were mutually exclusive. Cells expressing CBX3 tended to co‐express EMT marker (SNAI2) (Figure 9B,C). In histological study, the prepared tissue chip was stained with CBX3 and IL1RN. we here demonstrated the most representative pictures of CBX3^high^ and IL1RN^high^ tissues (Figure 9D). Across different tissue samples, high expression of CBX3 was associated with low expression of IL1RN, showing a negative correlation that is consistent with our previous findings (Figure 9E). In addition, colocalization analysis performed using ImageJ demonstrated a lack of colocalization between CBX3 and IL1RN (Figure S10A). Each sample was then labeled based on the relative expression score of CBX3:IL1RN. Although female patients were often IL1RN^high^, no clear sex bias was observed overall (Figure S10B). Age did not differ significantly between CBX3^high^ and IL1RN^high^ patients (Figure S10C). CBX3^high^ was more common in tongue carcinoma, while IL1RN^high^ was prevalent in buccal carcinoma (Figure S10D). Consistent with earlier studies, CBX3^high^ patients had higher grades than IL1RN^high^ patients (Figure S10E). Notably, in OSCC patients with T stage ≤ T2, approximately 30% of CBX3^high^ patients showed early lymph node metastasis (Figure S10G), suggesting they might benefit from early neck dissection. These findings indicated that CBX3 and IL1RN revealed distinct cellular states and pathways affecting patient survival.

## 4 Discussion

Patients with OSCC need to be stratified into biologically and clinically meaningful subtypes to better explain the heterogeneity in therapeutic and to enable more precise and personalized treatment strategies. However, the complexity of current transcription‐based methods, the burden of extensive gene signatures, and the lack of clinical guidance hinder their practical application in routine oncology settings.

In this study, we leveraged publicly available datasets to distinguish OSCC from CMS1 and CMS2 based on their molecular characteristics. CMS1 patients displayed significant mutations in TP53, EMT activation, abundant fibroblast infiltration, poor survival, and sensitivity to cisplatin and docetaxel, while CMS2 patients displayed activation of the inflammation pathway, good prognosis, and sensitivity to fluorouracil and PD‐1/PD‐L1 targeted therapy.

To further facilitate clinical translation, we used the TSP algorithm and identified the CBX3:IL1RN gene pair as a simple yet effective biomarker reflecting the CMS biological behavior, TME, and clinical features. Using our independent clinical samples, we further validated that CBX3 and IL1RN were negatively correlated, and the ratio of CBX3:IL1RN could further predict the clinical stage and likelihood of early lymph node metastasis.

The more valuable problem addressed here is the vulnerability of OSCC, which is able to directly assist neoadjuvant chemotherapy. Patients who responded to TPF therapy showed superior overall survival and locoregional and distant control [43, 44, 45]. Our findings illustrated that CMS1 patients were suitable for cisplatin and docetaxel therapy, whereas CMS2 patients were suitable for fluorouracil and PD‐1/PD‐L1 targeted therapy. Although CBX3:IL1RN showed unsatisfactory prediction of fluorouracil and cetuximab response, CBX3:IL1RN perfectly reflected the response to cisplatin and docetaxel. IL1RN^high^ patients could receive good survival simply by receiving standard surgery, and CBX3^high^ patients may benefit more from receiving both chemotherapy and surgery.

Mechanistically, both CMS markers and CBX3:IL1RN highlighted the activation of the EMT program, hinting at a potential mechanism that led to varied prognoses. Interestingly, our findings suggest that IL1RN in malignant cells and CBX3 in fibroblasts in the TME collectively led to the activation of EMT. In the spatial transcriptome, IL1RN was enriched in malignant cells that ranged along the basement membrane, whereas CBX3 was enriched in cells under these conditions. Immunofluorescence staining confirmed that IL1RN^+^ cells were surrounded by CBX3^+^ cells, in which CBX3^+^ cells resembled the edge of cancer. Above all, these findings were consistent with previous reports highlighting that the leading edge of cancer cells usually displays EMT activation through interaction with fibroblasts [5, 46].

CBX3 acts as a binding platform for histone modifiers that promote DNA methylation and is associated with epigenetic regulation, suggesting pro‐tumor effects [47, 48]. In contrast, IL1RN, a natural antagonist of IL‐1, serves as a negative regulator of IL‐1 and is considered an anti‐tumor factor in human squamous cell carcinoma (SCC) [49]. The imbalance between IL‐1 and IL1RN is thought to contribute to SCC development and metastasis [50]. Evidence shows that CBX3 and IL1RN are negatively correlated in various tissues and patients, yet the mechanisms behind this correlation in OSCC progression remain unclear. Our findings do not indicate genomic alterations in CBX3 and IL1RN in OSCC, but changes were observed in DNA methylation, transcription, and protein expression levels. Notably, CBX3 expression positively correlated with IL1RN methylation, suggesting that CBX3 may negatively regulate IL1RN through an epigenetic pathway, though further mechanistic validation is needed.

We identified the CBX3:IL1RN ratio as a key factor for prognostic prediction; however, several issues remain unresolved. Although the gene pair classifier determines subtypes by comparing intra‐individual gene expression, which mitigates batch effects, it cannot entirely avoid the residual effects caused by different platforms. Our analysis of bulk transcriptomic data revealed that the best cutoff for predicting survival varied across datasets and was not consistently CBX3:IL1RN = 1, likely due to differences in sequencing platforms and limited sample sizes. Additionally, while histological studies indicated that samples with high CBX3 and IL1RN levels exhibited clinical characteristics akin to CMS1 and CMS2, the appropriate threshold requires further validation before clinical implementation. Finally, we employed only a single gene pair, which may be insufficient to fully capture the complexity and heterogeneity within tumors. This gene pair system, however, enables a highly tractable clinical assay requiring only two measurements, which is a practical benefit for implementation. These challenges impede oncologists' and surgeons' decision‐making processes.

## Author Contributions

**Xutengyue Tian:** data curation (equal), formal analysis (equal), investigation (equal), methodology (equal), validation (lead), visualization (equal), writing – original draft (equal). **Jixiong Mao:** data curation (equal), formal analysis (equal), investigation (equal), methodology (equal), validation (equal), visualization (equal). **Dongguo Li:** data curation (equal), investigation (equal), methodology (equal). **Zhengxue Han:** conceptualization (equal), data curation (equal), funding acquisition (equal), methodology (equal), project administration (lead), resources (equal). **Qiaoshi Xu:** conceptualization (equal), data curation (equal), formal analysis (equal), funding acquisition (equal), investigation (equal), resources (lead), validation (supporting). Xutengyue Tian and Jixiong Mao contributed equally to this article.

## Funding

This study was supported by the Beijing Natural Science Foundation (7244350), the National Natural Science Foundation of China (82370925 and 82503857), and Beijing Stomatological Hosptial, Capital Medical University Young Scientist Program (YSP202111).

## Ethics Statement

Registry and registration no. of the study: The study was conducted according to the guidelines of the Declaration of Helsinki, and approved by the Institutional Review Board of the Beijing Stomatological Hospital of Capital Medical University (protocol code: CMUSH‐IRB‐KJ‐PJ‐2023‐25; date of approval: 12 May 2023).

## Consent

Informed consent was obtained from all enrolled patients.

## Conflicts of Interest

The authors declare no conflicts of interest.

## Supporting information


**Figure S1:** Quality control of single‐cell samples.
**Figure S2:** Identification of CMS1 and CMS2 as two subtypes for OSCC.
**Figure S3:** Distinction and stemness of malignant cells from CMS1 and CMS2 in multiple cohorts.
**Figure S4:** CMS classification implementation in multiple OSCC bulk transcriptomics.
**Figure S5:** Validation of construction and cell‐communication of CMS classification in an independent dataset.
**Figure S6:** CBX3:IL1RN is the best‐performing gene pair for classification.
**Figure S7:** Correlation analysis of the rest gene pair candidates.
**Figure S8:** CBX3:IL1RN proves clinical significance in multiple independent datasets.
**Figure S9:** CBX3:IL1RN mediates diverse cell‐communication networks between malignant cells and fibroblasts.
**Figure S10:** CBX3:IL1RN demonstrates clinical significance in multi‐immunofluorescence.

**Table S1:** Information of online available datasets.
**Table S2:** Clinical information of patients and samples.
**Table S3:** Robust CMS classification marker genes.

## Data Availability

The public datasets are available online. See Section 2 for details.
