# Supplementary material for: CBX3:IL1RN Reflects Distinct Cellular States That Defines the Clinical Outcome of Oral Squamous Cell Carcinoma
Source: Cancer Med. 2026 Mar 9;15(3):e71705. doi: 10.1002/cam4.71705 (PMC12971291; doi:10.1002/cam4.71705)
Supplement: Supplementary file 1 — Figure S1: Quality control of single‐cell samples. Figure S2: Identification of CMS1 and CMS2 as two subtypes for OSCC. Figure S3: Distinction and stemness of malignant cells from CMS1 and CMS2 in multiple cohorts. Figure S4: CMS classification implementation in multiple OSCC bulk transcriptomics. Figure S5: Validation of construction and cell‐communication of CMS classification in an independent dataset. Figure S6: CBX3:IL1RN is the best‐performing gene pair for classification. Figure S7: Correlation analysis of the rest gene pair candidates. Figure S8: CBX3:IL1RN proves clinical significance in multiple independent datasets. Figure S9: CBX3:IL1RN mediates diverse cell‐communication networks between malignant cells and fibroblasts. Figure S10: CBX3:IL1RN demonstrates clinical significance in multi‐immunofluorescence. [file CAM4-15-e71705-s001.docx]

**CBX3:IL1RN Reflects Distinct Cellular States that Defines the Clinical Outcome of Oral Squamous Cell Carcinoma**

*Xutengyue Tian, Jixiong Mao, Dongguo Li, Zhengxue Han^*^, Qiaoshi Xu^*^*


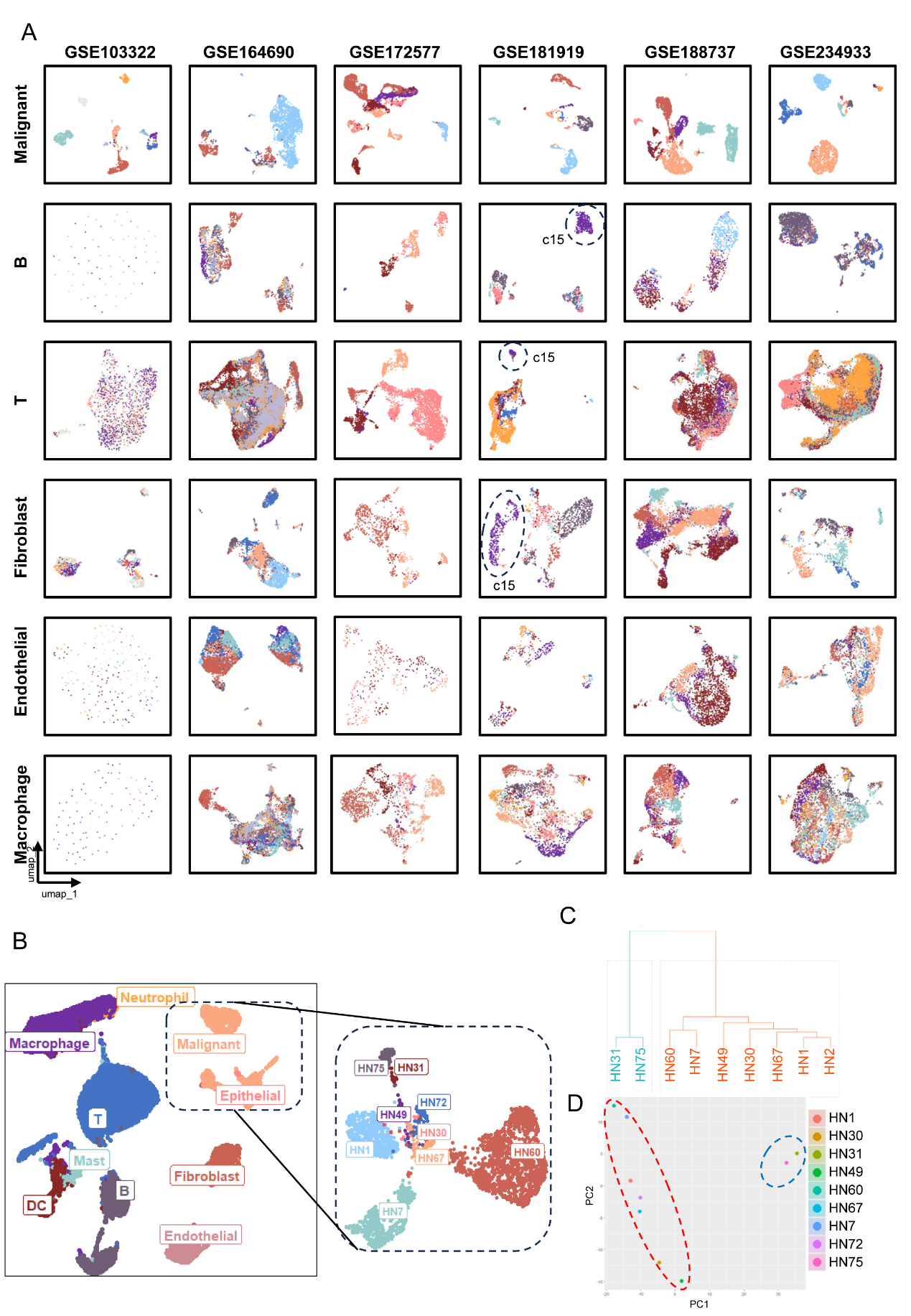


**Supplementary Figure 1.** Quality control of single-cell samples.

A) UMAP plot of malignant cells and non-malignant cells from 6 cohorts (patients are annotated). Samples only reveal specific clustering pattern in malignant cells are included. B) UMAP plot of GSE234933 OSCC patients. Major cell types are clustered and annotated (left). UMAP plot shows clustering of OSCC malignant cells retrieved from the 9 patients based on CMS classifier (right). C) Hierarchical plot displays pseudobulk data derived from OSCC malignant cells of the 9 patients, utilizing Euclidean distance as the metric and the average linkage method for clustering. D) PCA plot provides a visualization of the categorization of malignant cells from 9 patients.

**
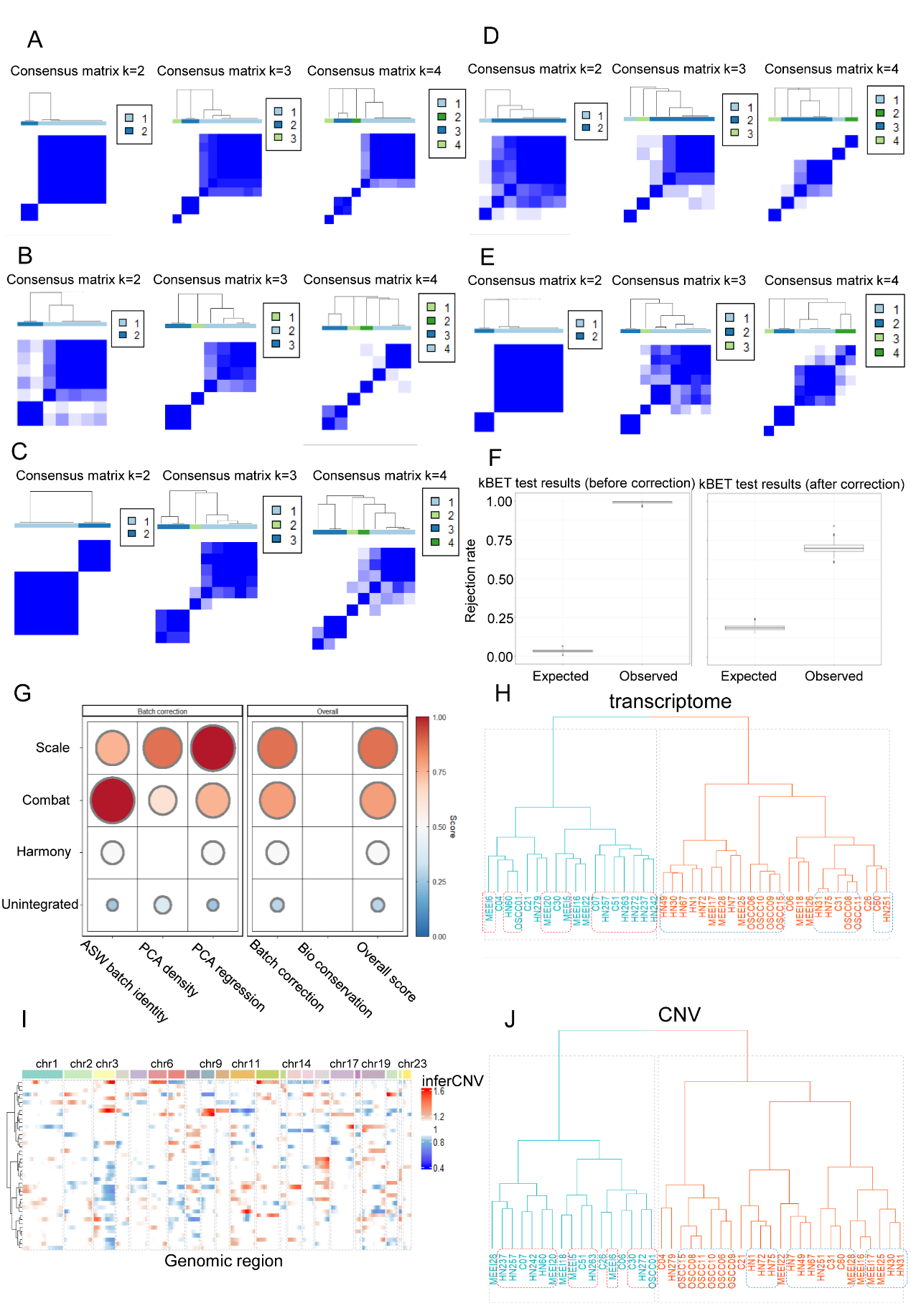
**

**Supplementary Figure 2.** Identification of CMS1 and CMS2 as two subtypes for OSCC.

A-E) Consensus clustering depicts the pseudobulk data for the datasets GSE103322, GSE164690, GSE181919, GSE188737, and GSE234933, respectively. F) Boxplot shows the results of kBET test comparing expected rejection rate and observed rejection rate before and after batch correction. H) Hierarchical plot illustrates the integrated bulk RNA transcriptomes, where the red square encompasses CMS1 patients, and the blue square encompasses CMS2 patients. G) The bubble chart demonstrates the score for each integration method (Scale, Combat, Harmony) and the unintegrated as control. The size and color of the bubble shows the score of ASW batch identity, PCA density, PCA regression, Batch correction, Bio conservation and Overall score (the closer to one, always the better). I) Heatmap of patient-specific pseudobulk CNV scores. Columns are lined based on chromosomal position of genes. Rows are clustered with hierarchical clustering. J) Hierarchical plot of integrated CNV profiles.

**
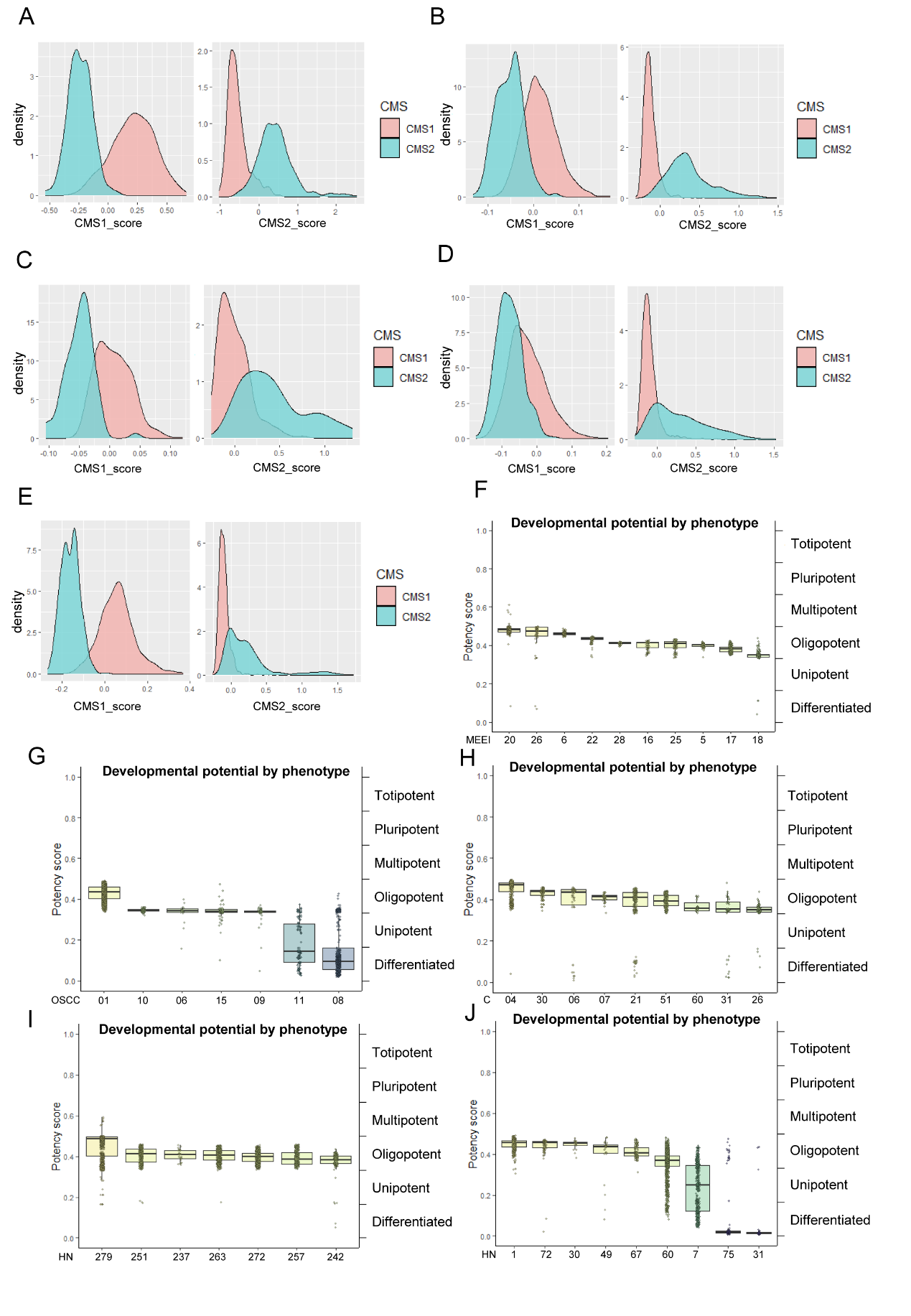
**

**Supplementary Figure 3.** Distinction and stemness of malignant cells from CMS1 and CMS2 in multiple cohorts**.** A-E) Density plots depict the distribution of module scores for datasets GSE103322, GSE164690, GSE181919, GSE188737, and GSE234933, based on the final markers specific to CMS1 and CMS2, respectively. F-J) Boxplots visualize the predicted potency scores obtained through Cytotrace for various patients across five single-cell datasets.

**
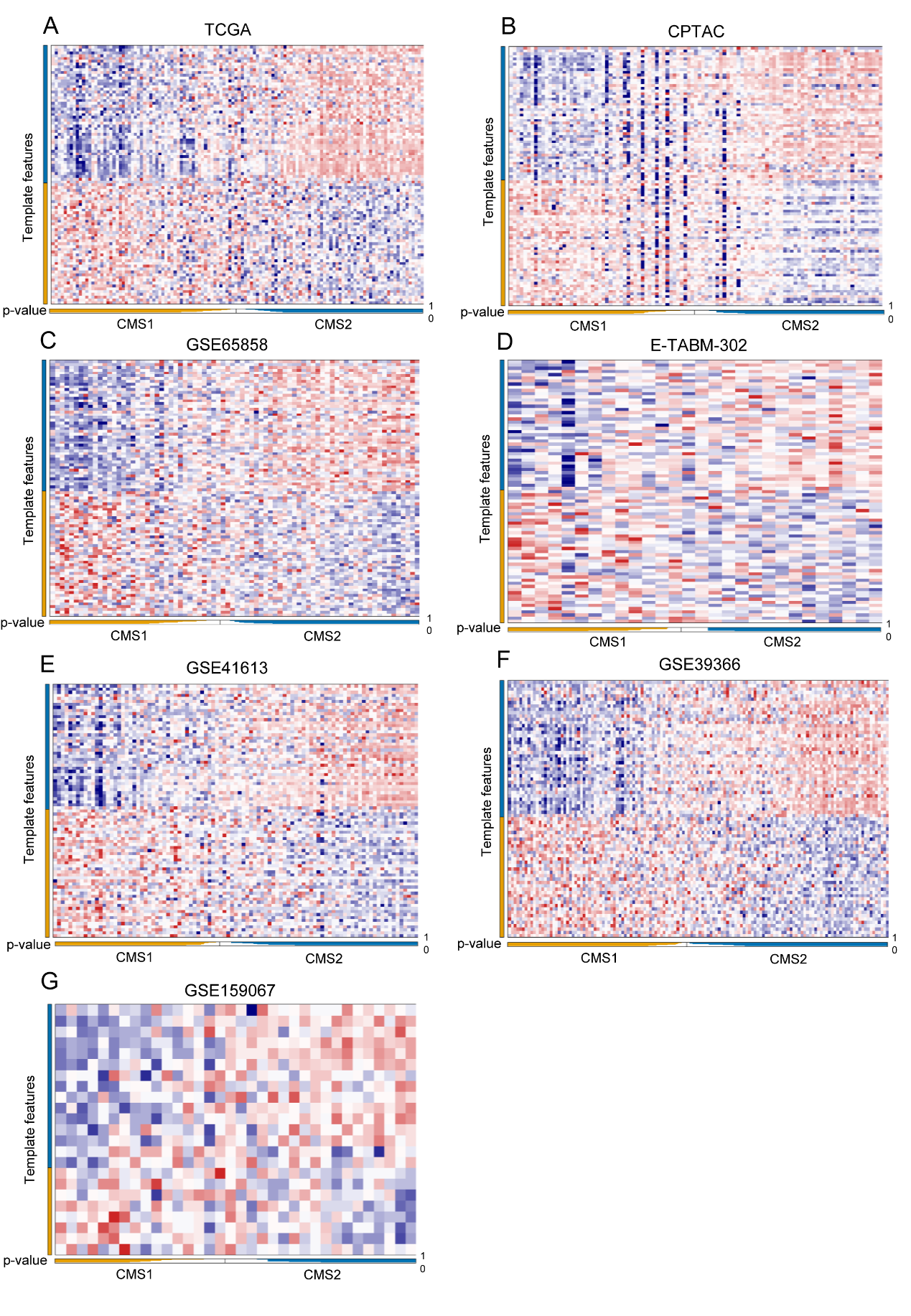
**

**Supplementary Figure 4.** CMS classification implementation in multiple OSCC bulk transcriptomics. A-G) Heatmaps of classified CMS1 and CMS2 based on the ntp algorithm in the CMScaller package across different datasets (Wilcoxon rank-sum test). Thresholds for p value is set as 0.05.

**
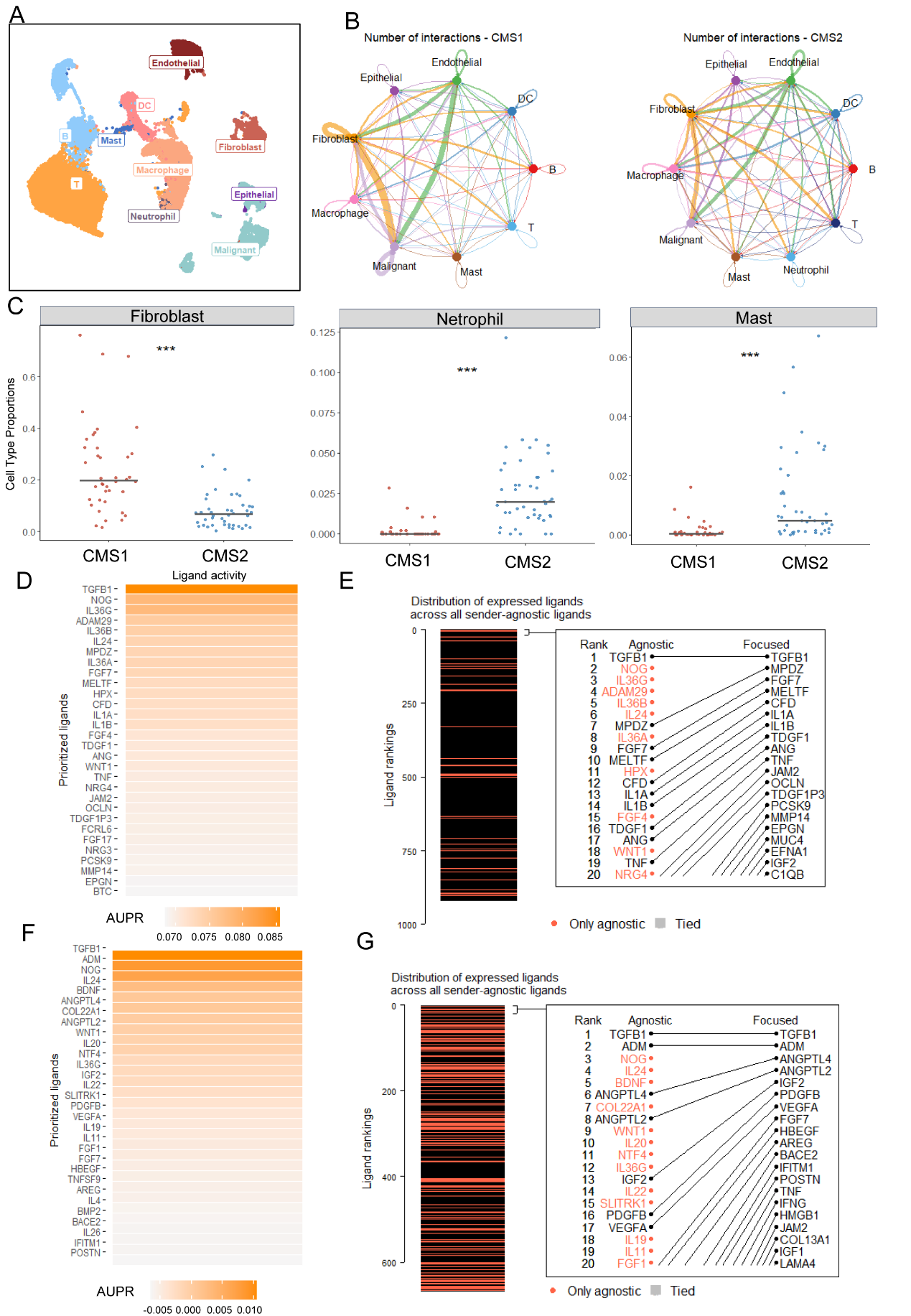
**

**Supplementary Figure 5.** Validation of construction and cell-communication of CMS classification in an independent dataset.

A) UMAP plot depicts patients from GSE234933 assigned to CMS classification, with annotations indicating the major cell types present. B) Chord plot displays the inferred number of interactions between CMS1 and CMS2. C) Plots visualize the enrichment of fibroblasts in CMS1, neutrophils and mast cells in CMS2 for validation, through integrated analysis of GSE234933 and TCGA (Student’s t test). Only cell types that p<0.05 are displayed. D-E) Plot illustrates the ranking of ligands within the ligand-receptor networks of CMS1 and CMS2, based on data derived from the CellChat dataset. F-G) Plots show ranked ligands of GSE234933 (Wilcoxon rank-sum test).


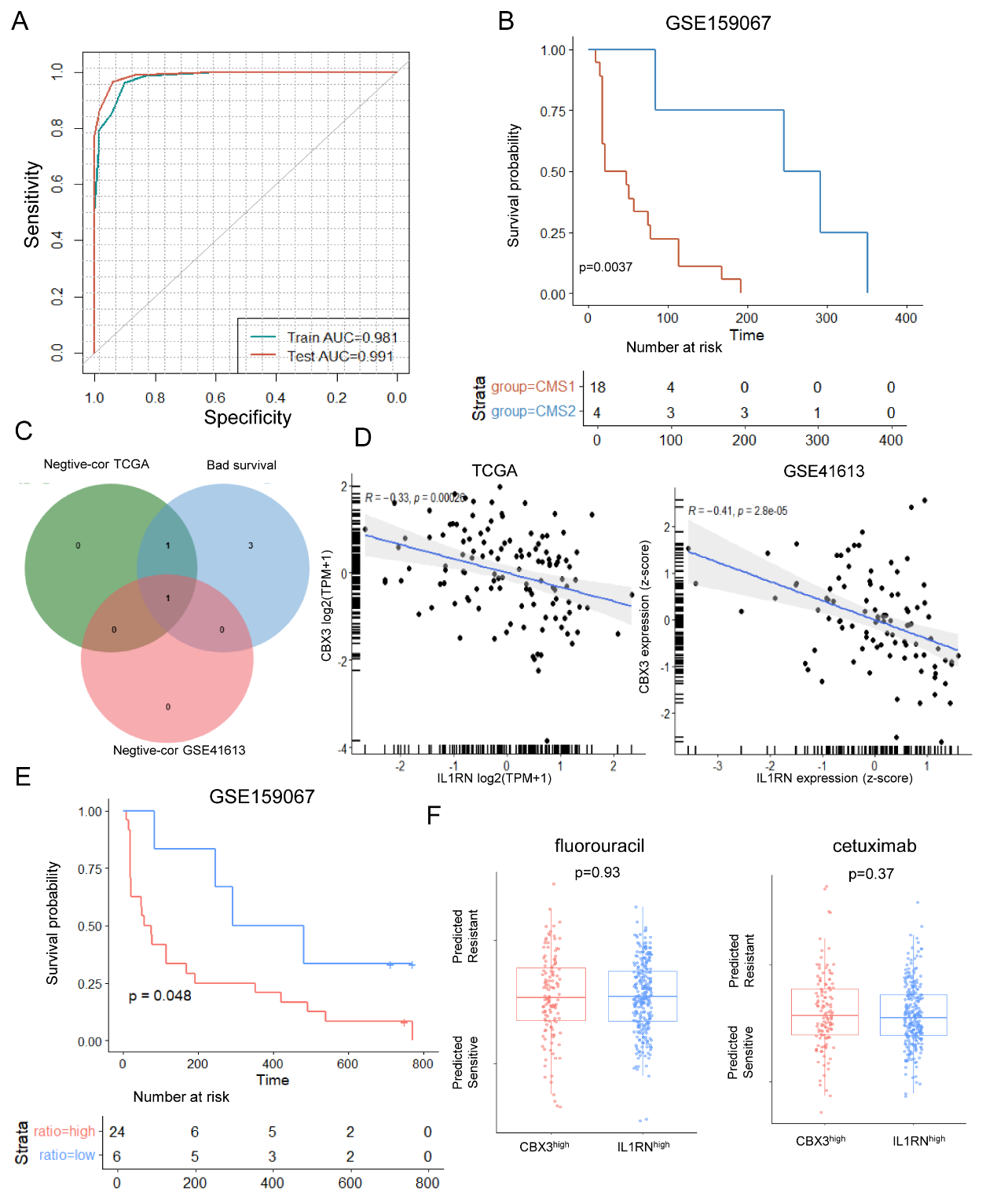


**Supplementary Figure 6.** CBX3:IL1RN is the best-performing gene pair for classification.

A) ROC curves presented validates the specificity and accuracy of both the CMS classification and the gene pair classification using data from TCGA and CPTAC. B) Kaplan-Meier curve depicts the survival analysis of CMS1 and CMS2 patients in the GSE159067 dataset, as classified by gene pair classification. C) Venn diagram illustrates the selection process for CBX3 and IL1RN. D) Scatter plot depicts a negative correlation between CBX3 and IL1RN expression levels in profiles from both TCGA (R=-0.33, p=0.00026) and GSE41613 (R=-0.41, p=2.8e-05) datasets. E) Kaplan-Meier survival curves validate the survival rates of patients with high CBX3:IL1RN ratio with low CBX3:IL1RN ratio in the GSE159067.F) Boxplots show the varied predicted chemosensitivity values to fluorouracil and cetuximab among CBX3^high^ patients and IL1RN^high^ patients (cutoff =1).

**
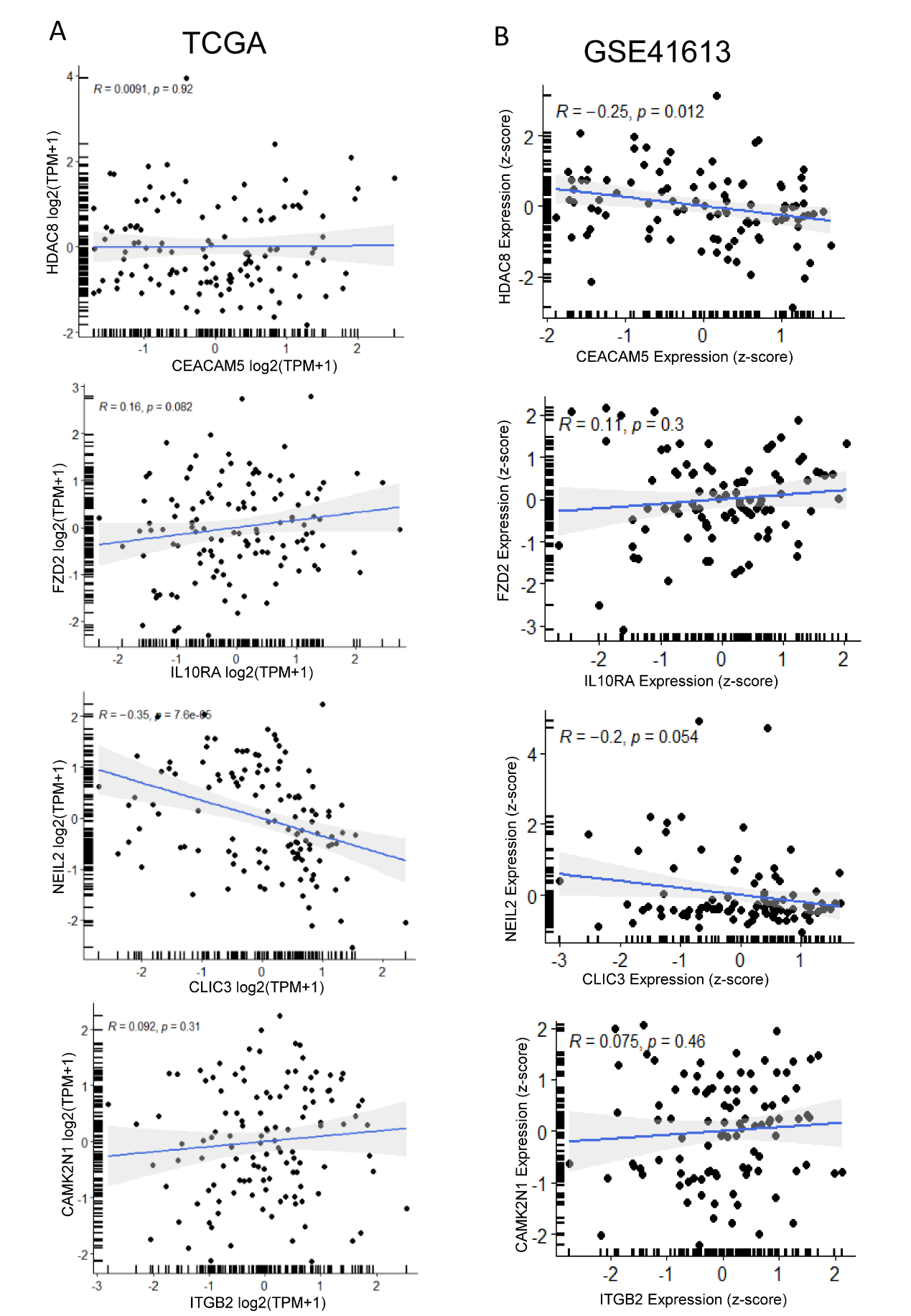
**

**Supplementary Figure 7.** Correlation analysis of the rest gene pair candidates

A) Scatter plots depict the spearman’s correlation of CEACAM5:HDAC8, IL10RA:FZD2, CLIC3:NEIL2, ITGB2:CAMK2N1 in TCGA dataset. Only CLIC3:NEIL2 displays a significant negative correlation (R=-0.36, p=7.6e-05). B) Scatter plots depict the spearman’s correlation of the rest gene pair candidates in GSE41613 dataset. Only CEACAM5:HDAC8 displays a significant negative correlation (R=-0.25, p=0.012).

**
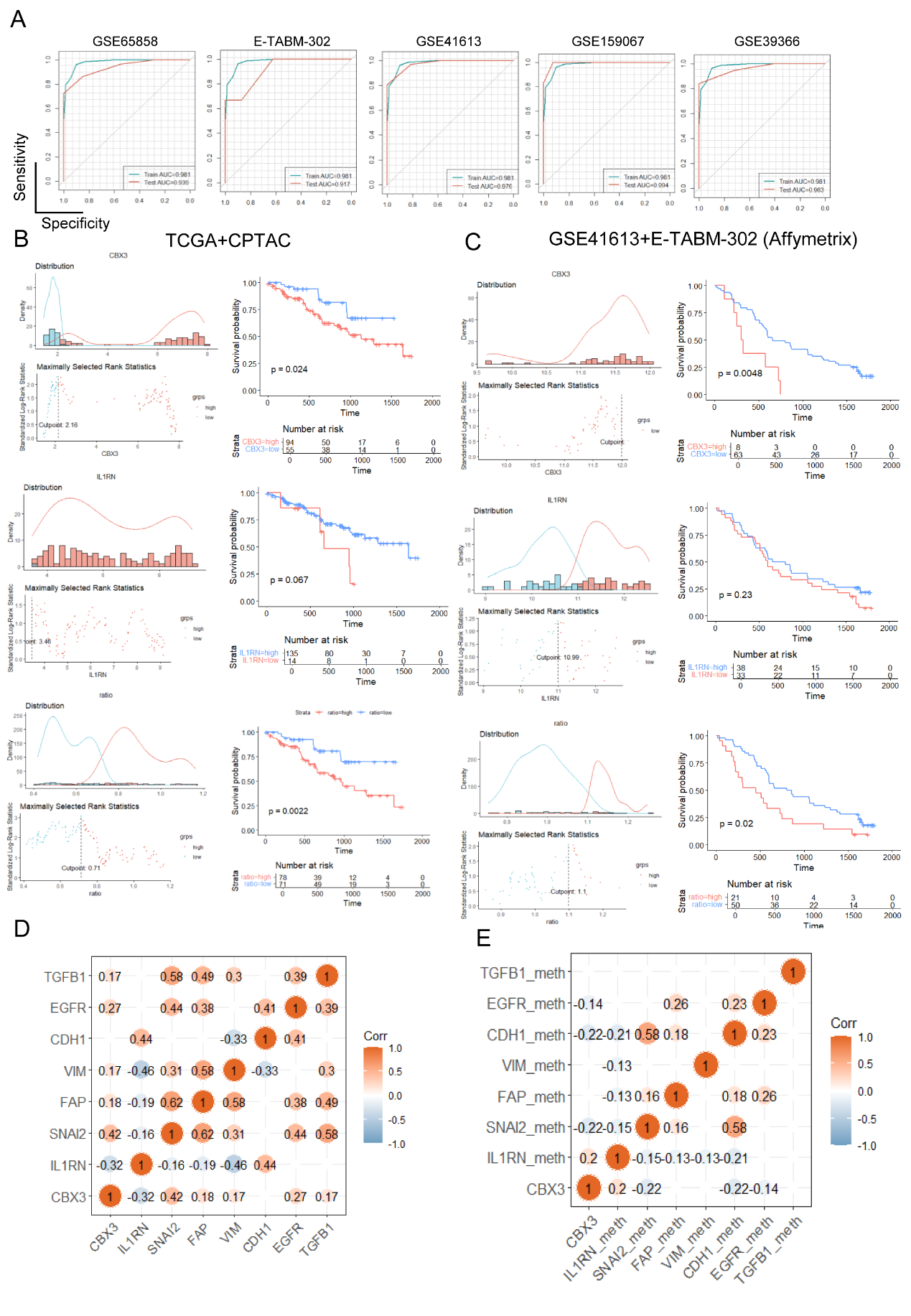
**

**Supplementary Figure 8.** CBX3:IL1RN proves clinical significance in multiple independent datasets. A) ROC plots validate the specificity and accuracy of the gene pair classification in distinguishing patients between CMS1 and CMS2 subtypes. B, C) Kaplan-Meier curves validate the effectiveness of CBX3, IL1RN and CBX3:IL1RN in predicting overall survival internally (TCGA+CPTAC) and externally (GSE51613+E-TABM-302). D) Correlation analysis of CBX3 with IL1RN, EGFR, and EMT-associated genes. E) Correlation analysis of CBX3 with methylation of IL1RN, EGFR, and EMT-associated genes.

**
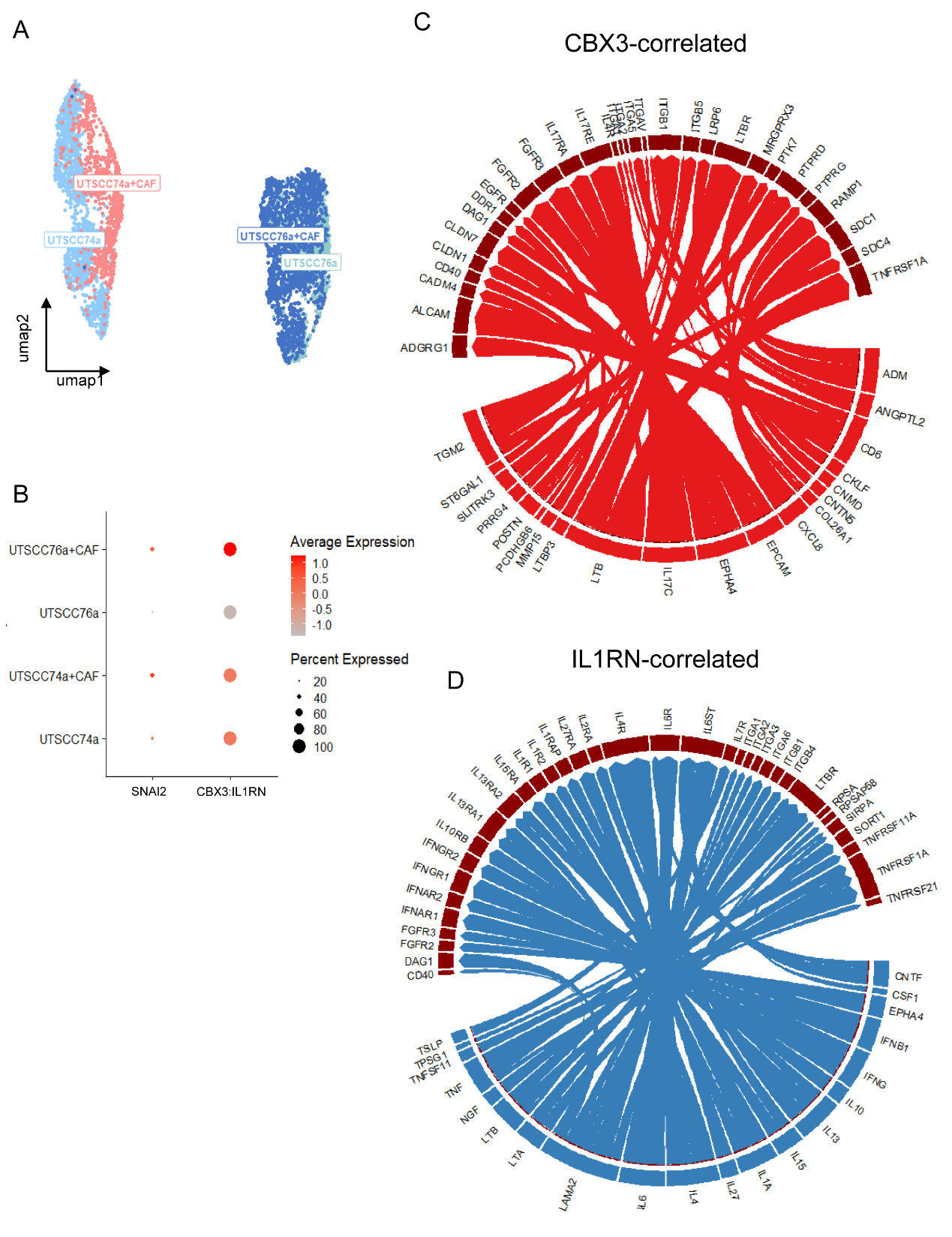
**

**Supplementary Figure 9.** CBX3:IL1RN mediates diverse cell-communication networks between malignant cells and fibroblasts.

A, B) UMAP plot of GSE251902. The groups and cell types are labeled. Dot plot shows SNAI2 expression in malignant cells with and without CAF co-culture. C, D) Circle plots demonstrate the patterns of CBX3-associated and IL1RN-associated ligand-receptor interactions between malignant cells and fibroblasts.

**
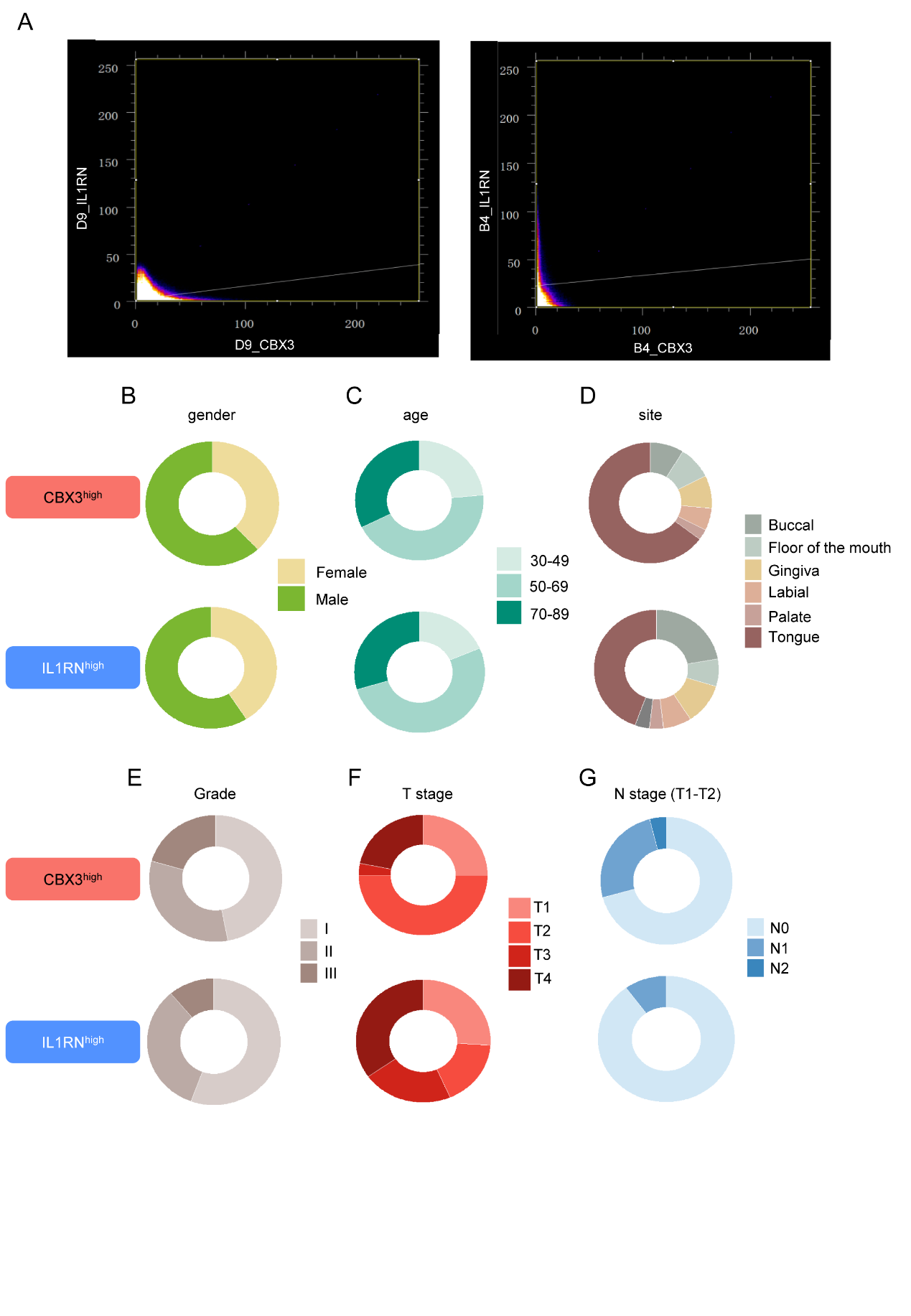
**

**Supplementary Figure 10.** CBX3:IL1RN demonstrates clinical significance in multi-immunofluorescence. A) Colocalization analysis of patients labeled with D9 and B4. The distribution of CBX3 and IL1RN in patients D9 and B4 exhibits no colocalization relationship. B) Pie charts show Clinical characteristics of CBX3^high^ and IL1RN^high^ patients, included gender, age, site, grade, T stage, and N stage (limited to patients in T1-T2).
