# Supplementary material for: CBX3:IL1RN Reflects Distinct Cellular States That Defines the Clinical Outcome of Oral Squamous Cell Carcinoma
Source: Cancer Med. 2026 Mar 9;15(3):e71705. doi: 10.1002/cam4.71705 (PMC12971291; doi:10.1002/cam4.71705)
Supplement: Supplementary file 2 — Table S1: Information of online available datasets. Table S2: Clinical information of patients and samples. Table S3: Robust CMS classification marker genes. [file CAM4-15-e71705-s002.docx]

**Supplementary table 1. Information of online available datasets**

| **Accession number** | **Description** | **Platform** | **Sample size (primary OSCC)** |
| --- | --- | --- | --- |
| GSE103322 | HPV- OSCC and metastases in lymph nodes (single-cell) | Smart-Seq2 | 10 |
| GSE164690 | HPV+/HPV- treatment naive HNSCC (single-cell) | 10x Genomics | 7 |
| GSE181919 | Normal tissue, leukoplakia, primary cancer and metastases in lymph nodes (single-cell) | 10x Genomics | 9 |
| GSE188737 | Primary HNSCC and metastases in lymph nodes (single-cell) | 10x Genomics | 7 |
| GSE234933 | Primary, local recurrent and distant metastasis HNSCC (single-cell) | 10x Genomics | 9 |
| GSE215403 | Primary gingiva-buccal OSCC (single-cell) | 10x Genomics | 12 |
| GSE251902 | Human tongue SCC co-cultured with CAFs (single-cell) | BD | 2 |
| TCGA-HNSC | HNSCC (bulk) | RNA-seq | 257 |
| CPTAC-HNSC | HNSCC (bulk) | RNA-seq | 105 |
| GSE65858 | Primary HNSCC (bulk) | Microarray (Illumina) | 78 |
| GSE41613 | HPV- primary OSCC (bulk) | Microarray (Affymetrix) | 97 |
| GSE39366 | Primary HNSCC (bulk) | Microarray (Agilent) | 55 |
| GSE159067 | PD-1/PD-L1 targeted HNSCC (bulk) | RNA-seq | 34 |
| E-TABM-302 | Primary HNSCC (bulk) | Microarray (Affymetrix) | 28 |
| E-MTAB-8588 | Primary HNSCC (bulk) | Microarray (Illumina) | 19 |
| GSE208253 | Primary OSCC (spatial) | 10x Genomics | 12 |

**Supplementary table 2. Clinical information of patients and samples**

| Number | Age | Gender | Site | T Stage | N Stage | Grade |
| --- | --- | --- | --- | --- | --- | --- |
| G0 | 63 | female | gingiva | 2 | 2 | 1 |
| G1 | 65 | female | tongue | 2 | 1 | 3 |
| G2 | 74 | female | palate | 2 | 0 | 3 |
| G3 | 83 | male | tongue | 1 | 0 | 1 |
| G4 | 82 | male | lower labial | 1 | 0 | 1 |
| G5 | 63 | female | lower labial | 2 | 0 | 1 |
| G6 | 67 | male | gingiva | 4 | 0 | 3 |
| G7 | 74 | male | tongue | 2 | 0 | 1 |
| G8 | 62 | male | tongue | 2 | 1 | 1 |
| G9 | 48 | male | tongue | 4 | 1 | 2 |
| G10 | 41 | male | tongue | 3 | 1 | 1 |
| F1 | 63 | male | tongue | 3 | 0 | 2 |
| F2 | 73 | male | tongue | 2 | 0 | 2 |
| F3 | 85 | female | labial | 1 | 0 | 1 |
| F4 | 54 | male | floor of the mouth | 3 | 0 | 2 |
| F5 | 59 | male | buccal | 1 | 0 | 1 |
| F6 | 75 | female | gingiva | 3 | 0 | 2 |
| F7 | 66 | male | buccal |  | 1 | 1 |
| F8 | 77 | female | gingiva | 1 | 0 | 1 |
| F9 | 37 | male | buccal | 4 | 1 | 3 |
| E1 | 68 | female | tongue | 2 | 0 | 1 |
| E2 | 50 | female | tongue | 4 | 0 | 1 |
| E3 | 44 | male | floor of the mouth |  | 1 | 1 |
| E4 | 58 | male | floor of the mouth | 4 | 0 | 2 |
| E5 | 81 | female | tongue and floor of the mouth | 2 | 0 | 1 |
| E6 | 45 | female | tongue | 2 | 1 | 2 |
| E7 | 39 | female | tongue | 1 | 1 | 1 |
| E8 | 68 | male | labial | 1 | 0 | 1 |
| E9 | 57 | male | tongue |  | 1 | 1 |
| E10 | 83 | female | buccal | 2 | 0 | 2 |
| E11 | 60 | female | buccal | 2 | 0 | 1 |
| D1 | 74 | female | tongue | 2 | 0 | 1 |
| D2 | 46 | female | tongue |  | 1 | 2 |
| D3 | 65 | male | tongue | 2 | 1 | 2 |
| D4 | 70 | male | tongue | 1 | 0 | 1 |
| D5 | 58 | female | tongue | 2 | 0 | 1 |
| D6 | 54 | male | floor of the mouth | 4 | 1 | 3 |
| D7 | 64 | female | tongue | 1 | 0 | 2 |
| D8 | 57 | male | tongue | 1 | 0 | 2 |
| D9 | 58 | male | tongue | 2 | 1 | 2 |
| D10 | 56 | male | tongue | 4 | 1 | 1 |
| D11 | 47 | female | tongue | 2 | 0 | 2 |
| D12 | 58 | male | retromolar |  | 1 | 1 |
| C1 | 54 | male | tongue | 1 | 0 | 2 |
| C2 | 55 | male | buccal |  | 1 | 2 |
| C3 | 76 | female | gingiva | 2 | 0 | 1 |
| C4 | 62 | male | tongue | 1 | 0 | 1 |
| C5 | 31 | female | tongue | 1 | 0 | 2 |
| C6 | 77 | female | tongue | 2 | 0 | 2 |
| C7 | 75 | male | buccal | 4 | 0 | 1 |
| C8 | 59 | male | gingiva | 4 | 1 | 1 |
| C9 | 49 | male | buccal | 4 | 0 | 1 |
| C10 | 76 | female | tongue | 3 | 1 | 1 |
| C11 | 75 | male | tongue | 4 | 0 | 3 |
| B1 | 76 | female | tongue | 3 | 0 | 3 |
| B2 | 65 | male | tongue | 1 | 0 | 3 |
| B3 | 42 | male | palate | 4 | 0 | 2 |
| B4 | 59 | male | floor of the mouth | 4 | 0 | 2 |
| B5 | 45 | male | tongue | 4 | 1 | 3 |
| B6 | 31 | male | buccal | 4 | 1 | 1 |
| B7 | 75 | male | tongue | 2 | 1 | 3 |

**T stage: 1 = T1, 2 = T2, 3 = T3, 4 = T4**

**N stage: 0 = N0, 1 = N1, 2 = N2**

**Grade: 1 = well differentiated, 2 = moderate differentiated, 3 = poorly differentiated**

**Supplementary table 3. Robust CMS classification marker genes**

| LINC01206 | CNPY4 | S100P | ETV5 | CALML5 |
| --- | --- | --- | --- | --- |
| IQCJ-SCHIP1 | SBSN | SPRR2D | TCF4 | COL4A2 |
| CHI3L2 | LRCH3 | ADAM12 | S100A7A | TRPM2 |
| TGM1 | RFC4 | KRT13 | BICD1 | TMPRSS11A |
| DNAJB11 | TRAM2 | FADS2 | DEFB1 | PRELID3A |
| MPP6 | CTSZ | SAA2 | IL1R2 | ASNS |
| SPRR2A | ABCC5 | SLC36A1 | MMP11 | CHRNA5 |
| MRGBP | AQP3 | S100A9 | PLOD3 | TFRC |
| TOPBP1 | PAQR3 | ALMS1 | ANKRD22 | GOLIM4 |
| HOXC8 | MTBP | LRP8 | CHL1 | C1orf167 |
| LSG1 | E2F7 | DPY19L1 | SPRR2F | ARTN |
| PDIA5 | TFDP2 | B3GNT9 | MAGEF1 | MKRN2OS |
| GNA13 | KATNAL1 | SYCP2 | SEMA4F | CSTB |
| NXPE3 | FAM219A | MYZAP | NCOA7 | HSD17B2 |
| SLC25A36 | SULF1 | FBXO17 | S100A7 | MIR200CHG |
| NT5DC4 | LCN2 | ZNF639 | CEACAM6 | LPAR6 |
| AL589182.1 | CENPJ | C3 | HOPX | S100A14 |
| IGLV2-14 | AC007326.4 | EXOC3L1 | CEP97 | AC233280.1 |
| KIF21A | MXRA7 | JUNB | FAM171B | CSRNP1 |
| YEATS2 | PHF3 | ITGB5 | SOD2 | PDZK1IP1 |
| CLCN2 | IL36G | C1orf216 | UNC119B | SPINK5 |
| KDELR3 | PLA2G4E | BACH2 | BIRC3 | UBD |
| BIRC2 | IER2 | PLEKHG4B | B4GAT1 | KRT7 |
| LRP12 | CRCT1 | POU2F3 | NFIX | CHST7 |
| SORL1 | OVOL1 | DLX6 | SPRR1B | SLC2A3 |
| S100A8 | MPP3 | TNFSF15 | CSTA | APOBEC3A |
| DENND2D | TOP1MT | PFN2 | TMEM44-AS1 |  |
